# C3G deregulation uncovers a dual role in B-cell lymphoma: tumor suppression and enhanced metastasis via Rap1 and Rac2 signaling

**DOI:** 10.1186/s12964-025-02551-y

**Published:** 2025-11-27

**Authors:** Alba Morán-Vaquero, Óscar Herranz, Ana Dávila-Hidalgo, Antonio Rodríguez-Blázquez, Cristina Fernández-Infante, Ignacio García-Tuñón, Elena Vuelta, Femke van der Meer, Coert Margadant, Carmen Guerrero, José M. de Pereda

**Affiliations:** 1 Centro de Investigación del Cáncer, Consejo Superior de Investigaciones Científicas (CSIC), Universidad de Salamanca, Campus Unamuno s/n, Salamanca, 37007 Spain; 2 Instituto de Investigación Biomédica de Salamanca (IBSAL), Salamanca, Spain; 3 Departamento de Medicina, Universidad de Salamanca, Salamanca, Spain; 4 Departamento de Biomedicina y Biotecnología, Universidad de Alcalá, Alcalá de Henares, Spain; 5 Institute of Biology, Leiden University, Gorleaus Laboratory, Einsteinweg 55, Leiden, 2333 CC The Netherlands

**Keywords:** C3G, B-cell lymphoma, Rap1 signaling, Rac2 signaling, Proliferation, Apoptosis, Adhesion, Migration, Metastasis

## Abstract

**Background:**

C3G (RapGEF1) is a guanine nucleotide exchange factor that activates Rap1, a small GTPase implicated in hematologic malignancies. We previously showed that C3G GEF activity is self-repressed via its AIR (autoinhibitory region). A lymphoma-associated missense mutation (Y554H) disrupts this inhibition, resulting in constitutive activation. This study aims to investigate the consequences of C3G dysregulation in B-cell lymphoma.

**Methods:**

Murine C3G mutation Y564H (equivalent to human Y554H) was introduced into the A20 B-cell lymphoma line using CRISPR/Cas9. Rap1 activation, proliferation, apoptosis, ERK1/2 phosphorylation, Rac2 activity, adhesion, migration, invasion, tumorigenicity, and transcriptomic changes were assessed through biochemical assays, in vitro functional studies, in vivo mouse models, and RNA-seq analysis.

**Results:**

A20-C3G-Y564H cells exhibited increased Rap1 activation under both basal and stimulated conditions. Hyperactivation of the C3G-Rap1 pathway impaired proliferation, promoted apoptosis, and was associated with reduced ERK1/2 phosphorylation. Furthermore, Rac2 activity was diminished, correlating with altered adhesion properties. Consistently, cell migration and invasion were enhanced, in correspondence with an increased number of metastatic foci in the liver following tail vein injection into syngeneic BALB/c mice. Notably, reduced C3G expression further augmented the metastatic potential of A20 cells. RNA-seq analysis revealed widespread transcriptional changes involving Rac2 signaling, adhesion, and metastatic pathways.

**Conclusions:**

C3G plays a dual role in B-cell lymphoma: it acts as a tumor suppressor by inhibiting growth and promoting apoptosis, but may also facilitate metastasis via enhanced motility. This dual effect likely reflects a functional balance between Rap1 and Rac2 signaling. These findings underscore the complexity of C3G-regulated pathways in B cells and suggest that C3G may serve as a potential novel marker in hematologic malignancies.

**Supplementary Information:**

The online version contains supplementary material available at 10.1186/s12964-025-02551-y.

## Abstract (plain language summary)

Understanding a key player in B-cell lymphoma: the surprising role of C3G

Scientists are uncovering how a protein called C3G, which helps control cell behavior, may play a complex role in a type of blood cancer known as B-cell lymphoma. Normally, C3G helps activate another protein called Rap1, but it keeps itself in check through a built-in “off switch.” However, a mutation found in some lymphoma patients disables this off switch, causing Rap1 to be constantly active. To better understand what this mutation does, researchers recreated it in mouse lymphoma cells using advanced gene-editing tools. They then studied how the mutated cells behaved in the lab and in mice. The results were surprising. The mutated cells showed more active Rap1, which led to slower growth and more cell death—typically signs of a weakened cancer. But at the same time, these cells became more mobile and invasive, spreading more easily to the liver when introduced into mice. This was linked to changes in another protein, Rac2, which helps cells stick together and move. By analyzing which genes were turned on or off in these cells, the team found major shifts in networks controlling cell movement and metastasis.

In short, C3G seems to have a double-edged role in lymphoma: it can help suppress tumor growth, but also make the cancer more likely to spread. These insights could open the door to new treatment strategies that target this delicate balance.

**Supplementary Information:**

The online version contains supplementary material available at 10.1186/s12964-025-02551-y.

## Background

C3G (RapGEF1) is a guanine nucleotide exchange factor (GEF) for members of the Ras and Rho families of GTPases, primarily targeting Rap1 (reviewed in [1]). The C3G-Rap1 axis plays a pivotal role in regulating cell-cell adhesion and cell interactions with the extracellular matrix (ECM) [2]. In addition, C3G regulates various physiological cellular processes, including proliferation, apoptosis, differentiation, actin cytoskeleton remodeling, and migration, through both Rap1-dependent and independent mechanisms [1]. C3G is also involved in malignant transformation, acting as a tumor suppressor or promoter depending on the cellular context [3]. Its downregulation has been associated with promigratory, invasive, and metastatic phenotypes in colorectal cancer (CRC) [4], hepatocellular carcinoma (HCC) [5], and glioblastoma (GBM) [6]. C3G also exhibits a suppressive effect on breast cancer [7]. In contrast, C3G upregulation has been linked to cancer progression in papillary thyroid carcinoma [8, 9], non-small cell lung cancer [10], as well as gastric and gynecological cancers [11, 12]. Additionally, hyperactivation of the C3G-Rap1 pathway has been implicated in the development of hematologic malignancies [13, 14].

Rap1 has been shown to antagonize Ras-ERK signaling by blocking c-Raf [15]. However, C3G can exert tumor suppressor functions independently of Rap1, through a distinct mechanism involving the activation of PP2A phosphatases, which dephosphorylate ERK1/2 [16–18].

C3G plays an essential role in embryogenesis primarily due to its crucial role in cell adhesion [19]. Its involvement in cellular differentiation has been reported across various cell types, including myocytes, adipocytes, neuroblasts [1], vascular endothelial cells [20], oval cells [21], megakaryocytes [22, 23], and embryonic stem cells [24]. In addition, C3G-Rap1 signaling is crucial for B-cell development [25, 26]. Activation of Rap GTPases is particularly important for B-cell morphological changes, which are likely linked to B-cell receptor (BCR)-induced integrin LFA-1 activation [27]. B cells with diminished levels of Rap1-GTP exhibit impaired migration toward C-X-C motif chemokine ligand 12 (CXCL12), a key chemoattractant [28]. Furthermore, Rap1 activation is essential for transendothelial migration of A20 cells, a process required for these mouse lymphoma cells to infiltrate tissues and form tumors in vivo [29]. Signaling through Rho family GTPases has also been linked to hematologic malignancies [30]. In particular, loss of Rac2, whose expression is restricted to hematopoietic cells [31], delays the onset of acute myeloid leukemia [32]. A crosstalk between Rap1 and Rac GTPases has been reported in several cell types, including fibroblasts [33] and pancreatic acinar cells [34]. In platelets Rac1 can be activated independently by both Rap1 and C3G [35, 36], while the reduced adhesion of Rac2^−/−^ B cells to ICAM1 correlates with decreased Rap1 stimulation [37].

Our previous research demonstrated that the GEF activity of resting C3G is self-repressed through an intramolecular interaction between an autoinhibitory region (AIR) and the catalytic Cdc25H domain [38]. Two missense mutations (Y554H and M555K) identified in non-Hodgkin lymphomas [39, 40] are located within the AIR, leading to constitutive activation of C3G [38].

In this study, we combined biochemical and cell biology techniques, as well as animal models, to investigate the impact of catalytic deregulation of C3G, specifically the C3G-Y564H mutation in mice (equivalent to the human C3G-Y554H mutation), on lymphoma cell biology. Our findings revealed that hyperactivation of the C3G-Rap1 pathway exerts a suppressive effect on lymphoma cell proliferation and survival by interfering with the Ras-ERK1/2 pathway. Furthermore, the C3G-Y564H mutation reduces cell adhesion to the ECM, likely through Rac2 inhibition, while promoting migration and invasion both in vitro and in vivo.

## Materials and methods

### Cell lines

Mouse Ba/F3 (RRID: CVCL_0161), WEHI-3B (RRID: CVCL_2239), and A20 (ATCC Cat# TIB-208, RRID: CVCL_1940) cells were grown in RPMI-1640 medium, supplemented with 10% fetal bovine serum (FBS), 2 mM L-glutamine, 100 U/mL penicillin, and 100 µg/mL streptomycin (all from Gibco). Interleukin-3 (IL-3)-dependent Ba/F3 cells were supplemented with 20% WEHI-3B-conditioned medium as a source of IL-3. A20 cell medium was further supplemented with 25 mM HEPES and 55 µM 2-mercaptoethanol (both from Gibco). Mouse Boff-p210 cells [14] were maintained in DMEM (Gibco) supplemented with 10% FBS, 2 mM L-glutamine, 100 U/mL penicillin, and 100 µg/mL streptomycin.

Primary human umbilical vein endothelial cell (HUVEC) pools from 3 to 5 individual donors were purchased from Lonza and cultured in endothelial growth medium-2 (EGM-2; Promocell), supplemented with 2 mM L-glutamine, 100 U/mL penicillin, and 100 µg/mL streptomycin (all from Sigma-Aldrich). HUVECs were used between passages 3 and 6, and cultured in tissue culture flasks coated with 0.1% (w/v) gelatin (Sigma-Aldrich).

All cells were cultured at 37 °C, 5% CO_2_, and 95% humidity.

### Genome editing with CRISPR/Cas9

The genome of Ba/F3, Boff-p210, and A20 cells was modified using the CRISPR/Cas9 editing methodology. Endogenous C3G expression was disrupted by knock-out (KO) in Ba/F3 and Boff-p210 cell lines. Additionally, the CRISPR/Cas9 system was used to introduce the C3G-Y564H point mutation (equivalent to the human C3G-Y554H mutation [38]) into the endogenous gene via knock-in (KI) in A20 cells.

Genomic engineering reagents were introduced into the cell lines by electroporation, using the Neon transfection system (Invitrogen). For that, low-passage cells (~ 3 × 10^5^ per condition) were electroporated with one pulse at 1700 V for 20 ms in cell media without antibiotics using the Alt-R CRISPR/Cas9 system (Integrated DNA Technologies), containing Alt-R Cas9 enzyme and the specific guide RNA (gRNA). The gRNA was generated by annealing an equal concentration of Alt-R CRISPR/Cas9 crRNA and Alt-R CRISPR/Cas9 tracrRNA-ATTO-550. The crRNAs used in this work were: 5´-GCGCTACTTTAAGACCATTG*TGG*−3´, which aligns in C3G exon 4 and was used to disrupt it, and 5´-GTCCTCCAGCAGTTGCAT**GTA***GG*−3´, in exon 11, including the Y564 codon (in bold). The protospacer adjacent motif (PAM) sequence in both oligos is underlined. In the last case, a single-stranded oligodeoxynucleotide (ssODN) (4 µM) was additionally incorporated to introduce the point mutation of interest (C3G-Y564H) by Homology-Directed Repair (HDR) in A20 cells and a NdeI restriction site (underlined). The sequence of the ssODN was 5’- GCTCCTCGGGGGCCCCAAGAACACGAGAGCTGGTGGGGACAGCTCTGTGGGCCCCACACTGGTGAGATAGCAGCCCCCTCTGTCCACAGTGCTGGC**A***CATATG*CAACTGCT**A**GAGGACTACTCAGAGCCACAGCCCTCCATGTTCTACCAGACACCGCAGAGTGAGCACATCTACCAGCAGAAGAACAAGATGCTCAT-3’. To improve the editing efficiency of HDR, 30 µM Alt-R HDR enhancer was added to the mixture. Single PAM-silent mutations that prevent further cleavage of the template are indicated in bold and boxed. As control, cells were electroporated only with Cas9 protein. Twenty-four hours post-electroporation, the ATTO-550 fluorescent reporter allowed the separation of individual cells through Fluorescence-Activated Cell Sorting (FACS), using a FACSAria III cytometer (BD Biosciences, RRID: SCR_016695). Cells were seeded into 96-well plates to establish single-cell clones.

### Analysis of genomic DNA of edited clones

Genomic DNA (gDNA) from expanded single-cell clones was extracted using the NZY Tissue gDNA Isolation Kit (NZYtech). The DNA sequence of interest was then amplified by PCR using primers C3G-KO forward (5´-TTGGTCCCTGCTGCCAAAGC-3´) and C3G-KO reverse (5´-AGCTGATCACAGGATAAAGCCA-3´) to analyze C3G disruption, and C3G-Y564H-KI forward (5´-GCATGTGTCCACCTTTGTAGTA-3´) and C3G-Y564H-KI reverse (5´-TAGGGTTCCACCACGGATAA-3´), followed by NdeI digestion, to analyze the point mutation. The amplified DNAs positive for NdeI were sequenced using an ABI-3130xl sequencer (Applied Biosystems).

### Generation of A20 cells with ectopic expression of the human C3G-Y554H mutant

A20 cells were electroporated with the pEF1-C3G-Y554H-EGFP construct [38], or with the corresponding empty vector (pEF1-EGFP). GFP-positive clones were selected by FACS and maintained in complete medium supplemented with 400 µg/ml G418.

### Western blotting

Proteins were separated by Tris-glycine SDS–polyacrylamide gel electrophoresis (PAGE) and transferred onto Immobilon-P membranes (Millipore). Primary antibodies were used at 1:1000 dilution unless otherwise indicated (Additional file 1: Table S1). Secondary antibodies were used at 1:5000 or 1:10000 dilution (Additional file 1: Table S2). Signals of DyLight secondary antibodies were detected by infrared fluorescence using an Odyssey Imaging System (LI-COR Biosciences, RRID: SCR_023765) and were quantified using FIJI Software (RRID: SCR_002285) [41]. Horseradish peroxidase (HRP)-conjugated secondary antibodies were detected by enhanced chemiluminescence (ECL) using ECL Detection Reagents (Cytiva) and Super RX-N films (Fujifilm); films were scanned using an Epson V700-Photo scanner, and the intensities of the bands were quantified using FIJI.

### GTPase activation assays by pull-down

The activation of GTPases Rap1, Rac1, Rac2, Cdc42, and Ras in A20 cells was analyzed by pull-down assays, as described previously [38], using GST-RalGDS-RBD for Rap1, GST-PAK-PBD for Rac1/2 and Cdc42 (a gift from Dr. X.R. Bustelo, IBMCC, Salamanca, Spain), and GST-Raf-RBD for Ras (a gift from Dr. E. Santos, IBMCC, Salamanca, Spain). Active and total proteins were detected by western blot with specific antibodies (Additional file 1: Table S1). In all pull-down assays active GTPase levels (GTP-bound) in each A20-C3G-Y564H clone were compared to the average levels in A20-C3G-WT cells, which also served as the normalization reference.

### Stimulation of A20 cells

A20 cells were stimulated with 10 µg/mL anti-IgM antibody clone RMM-1 (Biolegend, RRID: AB_315052) to activate the BCR, 100 ng/mL recombinant mouse CXCL12 to activate the C-X-C chemokine receptor type 4 (CXCR4), or 10 ng/mL phorbol 12-myristate 13-acetate (PMA) to activate the protein kinase C (PKC) pathway.

### PP2A activity assay

PP2A phosphatase activity was quantified using the “PP2A Immunoprecipitation Phosphatase Assay Kit” (Merck), according to the manufacturer’s instructions and [18]. This method measures PP2A activity by assessing dephosphorylation of a phospho-threonine peptide (K-R-pT-I-R-R).

### Analysis of apoptosis

Apoptosis was monitored in Ba/F3 and Boff-p210 edited cells using the Annexin V-DY634 Apoptosis Detection Kit (Immunostep), following manufacturer’s instructions. Briefly, 5 × 10^5^ cells were incubated in Annexin V buffer (10 mM HEPES-NaOH pH 7.4, 140 mM NaCl, 25 mM CaCl_2_) with DY634-Annexin V and propidium iodide (PI) for 15 min at room temperature in the dark. Samples were processed by flow cytometry using a FACSCalibur cytometer (BD Biosciences, RRID: SCR_000401). In the case of A20 cells, apoptosis was measured using the FITC-Annexin V Apoptosis Detection Kit with 7-amino-actinomycin D (7-AAD, Immunostep), following manufacturer’s instructions. After labeling, samples were processed by flow cytometry using a BD Accuri C6 + cytometer (BD Biosciences, RRID: SCR_019591).

### Cell cycle assay

Cell cycle analysis was performed as in [22]. Briefly, Ba/F3 and Boff-p210 edited cells (5 × 10^5^ cells) were permeabilized in ice-cold 70% ethanol at 4 °C for 30 min. Then, cells were incubated in 500 µL PBS with 25 µg RNase and 7.5 µg PI, and DNA content was measured in a FACSCalibur cytometer (BD Biosciences).

### Expression analysis of endogenous surface proteins in A20 cells

Cell surface expression levels of CXCR4 (CXCL12 receptor), lymphocyte function-associated antigen 1 (LFA-1) and very late antigen-4 (VLA-4) integrins were simultaneously quantified by flow cytometry. Steady-state A20 cells (~ 10^6^) were harvested and washed in cold PBS supplemented with 5% FBS. Subsequently, ~5 × 10^5^ cells were resuspended in 50 µL of PBS with 0.5% FBS and PE anti-mouse CXCR4 (1:50) from R&DSystems (RRID: AB_2230502), APC anti-mouse CD11a (LFA-1 α chain) (1:100) (RRID: AB_2562778), and PerCP/Cyanine5.5 anti-mouse CD49d (VLA-4 α chain) (1:50) (RRID: AB_2563701) (both from BioLegend). Incubation was performed for 20 min on ice in the dark. After washing, cells were resuspended in 200 µL of cytometry buffer (PBS with 0.2% BSA and 2 mM EDTA) prior to acquisition using a BD Accuri C6 + cytometer (BD Biosciences). Data are presented as mean fluorescence intensity (MFI), which refers to the average brightness of each individual event.

### Cell viability by MTT assay

This colorimetric assay measures the reduction of the yellow tetrazolium dye MTT (Merck) to purple formazan crystals by metabolically active cells [42]. Approximately 2 × 10^4^ A20 cells were seeded in triplicate into 96-well plates at 100 µL per well in complete medium and analysis was performed at 24, 48, 72, and 96 h. MTT (0.5 mg/mL) was added at the indicated time points and the cells were incubated for 4 hours at 37 °C in a 5% CO_2_​ environment, protected from light. After MTT reduction, the plate was centrifuged at 445 x*g* for 5 min to precipitate insoluble formazan crystals, which were then solubilized in 100 µL DMSO by gently shaking for 10 min. Absorbance at 570 nm was measured using an Infinite M200 Pro plate reader (TECAN, RRID: SCR_019033).

### Cell proliferation by flow cytometry

A20 cell proliferation over time was measured by flow cytometry. Cells (~ 1.5 × 10^5^) were seeded in triplicate in 1 mL RPMI in 24-well plates. Four plates were prepared simultaneously for the analysis at four different time points (6, 24, 48, and 72 h). At each time point, cells were harvested and incubated with PBS containing 1% FBS and FITC-anti-mouse/human CD45R/B220 antibody (1:200) from BioLegend (RRID: AB_312990) for 20 min on ice in the dark. The total number of cells was quantified by acquisition of a constant volume in a BD Accuri C6 + cytometer (BD Biosciences). Live cells were identified based on FSC/SSC parameters and FITC positivity.

### Cell proliferation by live-cell imaging microscopy

To analyze cell proliferation cultures in real-time, approximately 2 × 10^4^ A20 cells were seeded in triplicate into 96-well plates pre-coated with 50 µL of 0.01% poly-L-ornithine solution (Sigma-Aldrich) for 1 h at room temperature. After seeding, cells were allowed to settle for 30 min at room temperature. The plate was then transferred to an Incucyte^®^ SX5 instrument (Sartorius, RRID: SCR_026298) and equilibrated at 37 °C for ~ 1 h before initiating scans. Four images per well were captured every 6 h over a 96-hour period using the phase-contrast channel and a 20X objective. Data acquisition and analysis were performed using the Non-Adherent Cell-by-Cell Analysis software module (Sartorius), and a 4-parameter Gompertz growth model was fitted to the resulting data.

### Cell adhesion assay

Static adhesion assays were conducted using 96-well plates with a non-treated surface (ThermoFisher), coated with 2 mg/mL human fibrinogen or 20 µg/mL fibronectin from bovine plasma (both from Sigma-Aldrich) for 2 h at room temperature. After coating, excess material was removed by washing with PBS. To block non-specific binding sites, the wells were incubated with 1% BSA in PBS for 1 h at room temperature. Approximately 5 × 10^5^ starved A20 cells, either untreated or stimulated with 20 nM freshly-prepared PMA for 10 min at 37 °C, were seeded in 150 µL of complete medium per well. Cells were allowed to adhere to the substrate-coated plates for 4 h at 37 °C in a 5% CO_2_​ environment. Non-adhered cells were removed and the remaining adhered cells were fixed with ice-cold methanol for 10 min at room temperature. Adhered cells were stained with crystal violet and relative absorbance at 550 nm, which is proportional to the number of cells, was measured using an Infinite M200 Pro plate reader (TECAN).

### Quantification of A20 cell adhesion to HUVECs

HUVECs were seeded in 96-well plates (5 × 10^3^ cells/well) and grown to confluence during 4 days, whereafter they received fresh EGM-2 without or with 10 ng/mL tumor necrosis factor alpha (TNF-α) overnight to stimulate ICAM and VCAM expression. The next day, A20 cells were collected by centrifugation, resuspended in EGM-2 medium (1 × 10^6^/mL), and 2 × 10^5^ cells were seeded on top of the HUVEC monolayers and allowed to adhere for 60 min at 37 °C. Thereafter, non-adherent cells were washed away with PBS (with Ca^2+^ and Mg^2+^), and HUVEC monolayers with adherent A20 cells were fixed with 4% paraformaldehyde. Images were acquired on an EVOS Cell Imaging system (ThermoFisher) at 4X magnification (6 wells/condition in each experiment). Images were then processed in FIJI using the Labkit plugin. A classifier was trained to segment and recognize adherent A20 cells on binary images after watershedding, whereafter the ‘Analyze particles’ function was used for counting. The experiment was performed at least 3–4 times with essentially similar results. Data are expressed as the number of adherent cells relative to the adhesion of A20-C3G-WT cells on HUVECs without TNF-α.

### Cell migration and invasion assays

Approximately 10^5^ steady-state A20 cells were seeded into the upper chamber of a 5 μm polycarbonate Transwell insert (Corning) in 100 µL of complete medium containing 5% FBS. For the migration assay, the lower chamber was filled with 600 µL of complete medium supplemented with 200 ng/mL CXCL12 and 10% FBS to induce chemotaxis. As a negative control, the lower chamber contained medium with 5% FBS but without CXCL12. Migration was allowed to proceed for 48 h at 37 °C in a 5% CO_2_​ incubator. To account for potential cell proliferation during the incubation period, the same number of cells was plated in a separate well without an insert to assess the total number of live cells after 48 h. The number of cells that migrated to the lower chamber was quantified using a Neubauer chamber and normalized to the total number of viable cells.

For the invasion assay, the Transwell inserts were pre-coated with 12 µg of 36 µg/cm² Matrigel (Corning), diluted in 100 µL of H_2_​O, and dried for over 14 h prior to cell seeding.

### Gene expression by RT-qPCR

Total RNA from A20 cells (~ 5 × 10^6^) was extracted with the RNeasy Mini Kit (Qiagen). Gene expression was determined by RT-qPCR reaction using First-Strand cDNA Synthesis kit, to produce cDNA, followed by NZYSpeedy qPCR Green Master Mix (both from NZYtech). Primers specific for the murine genes of interest are described in Additional file 1: Table S3. Each experiment was performed in triplicate, and β-actin (*Actb*) was used as housekeeping gene. qPCR reactions were prepared in either 96- or 384-well plates, and fluorescence was measured using Applied Biosystems QuantStudio 3 or QuantStudio 5 systems (ThermoFisher, RRID: SCR_018712 and RRID: SCR_020240), respectively. mRNA expression levels were calculated according to the 2^−ΔΔCt^ method [43].

### Transcriptome sequencing of A20 clones

Total RNA was extracted from A20 cells, as described above, for transcriptome analysis. Twelve independent samples were analyzed: two each from A20-C3G-WT clones 1, 2, and 3 (pooled as the A20-C3G-WT control), and three each from A20-C3G-Y564H clones 195 and 221. RNA integrity was assessed using the Bioanalyzer 2100 system (Agilent Technologies). mRNA libraries were prepared using poly-A enrichment. RNA sequencing was performed by Novogene on an Illumina NovaSeq 6000 platform (RRID: SCR_016387) using the NovaSeq PE150 pipeline. Paired-end 150 bp reads were generated and aligned to the *Mus musculus* reference genome (GRCm39) using Hisat2 v2.0.5. The number of counts mapped to each gene was obtained with featureCounts [44]. Differential expression analysis of biological replicates was performed using the DESeq2 v.1.20.0 package (RRID: SCR_015687) [45] in R, using raw counts as input. The DESeq function was applied to compare gene expression between WT and each mutant clone. Genes were considered significantly differentially expressed if they had a false discovery rate (FDR)-adjusted p-value ≤ 0.05 and |log_2_(fold_change)|≥1. Genes with similar expression patterns across samples were clustered and visualized in a heatmap, where red indicates high expression and green indicates low expression. Differentially expressed genes (DEGs) were further analyzed for functional enrichment using ClusterProfiler v3.8.1 (RRID: SCR_016884) [46]. Pathway enrichment was conducted using the Reactome database (RRID: SCR_003485), which integrates various reactions and biological pathways of human model species. Reactome pathways with an FDR-adjusted p-value ≤ 0.05 were considered significantly enriched.

The raw sequencing reads have been deposited in the European Nucleotide Archive (ENA) under accession number PRJEB90154.

### A20 cell injection into syngeneic BALB/c mice

Non-genetically modified BALB/c mice were purchased from Charles River Laboratories. For metastasis studies, ten- to twelve-week-old BALB/c mice were injected with 0.5 × 10^6^ A20 cells in 200 µL PBS via the tail vein. Animals were sacrificed no later than 21 days after injection, and the liver and spleen were dissected for metastasis analysis.

### Protein extraction from liver

At necropsy, small liver sections were excised and directly preserved by immersion in dry ice. Cytosolic proteins were extracted using the gentleMACs Dissociator (Miltenyi Biotec, RRID: SCR_020267) with M tubes and the Protein_01 program, following manufacturer’s instructions. Tissue homogenization and cell lysis were carried out in RIPA buffer containing 100 mM Tris-HCl pH 7.5, 100 mM NaCl, 1% Triton X-100, 1 mM EDTA, 0.1% sodium deoxycholate, 0.1% SDS, 1 mM Na_3_VO_4_, 25 mM NaF, 1 mM PMSF, and 1X cOmplete protease inhibitor cocktail (Roche).

### Analysis of tumor growth in liver and spleen

At necropsy, the liver and spleen of each mouse were dissected and immersed in cold PBS containing 1% FBS. For macroscopic analysis of tumor growth, organ weights were quantified and photographs were taken with a measurement calibration. Organs were then fixed by immersion in 4% formaldehyde for at least 24 h, and embedded in paraffin. Sections were stained with hematoxylin and eosin (H&E). Images of tissue sections were captured using an upright light microscope Leica DM 6 B (RRID: SCR_024857) with 5X objective and AmScope digital camera (model MU1803-CK-3PL), in combination with AmScope software. Images were processed with FIJI software [41].

### Statistical analysis

Unless otherwise specified, all the results are presented as the mean ± standard error of the mean (SEM) from at least three independent experiments. Statistical analyses were performed using GraphPad Prism v8.0.1 (RRID: SCR_002798). Data normality was assessed using the Shapiro-Wilk test. Differences between groups were evaluated with one-way or two-way ANOVA, depending on whether one or two factors were analyzed. In cases where missing values were present in the two-way ANOVA, a mixed-effects model was applied. For analyses involving A20-C3G-Y564H cells, the average of the three A20-C3G-WT clones was used as the control. ANOVA tests were followed by Sidak post-hoc test. The choice of statistical test was determined by data normality and homoscedasticity. P-values ≤ 0.05 were considered statistically significant.

## Results

### Deletion of C3G impairs the viability of pro-B cell lines

**Fig. 1:**
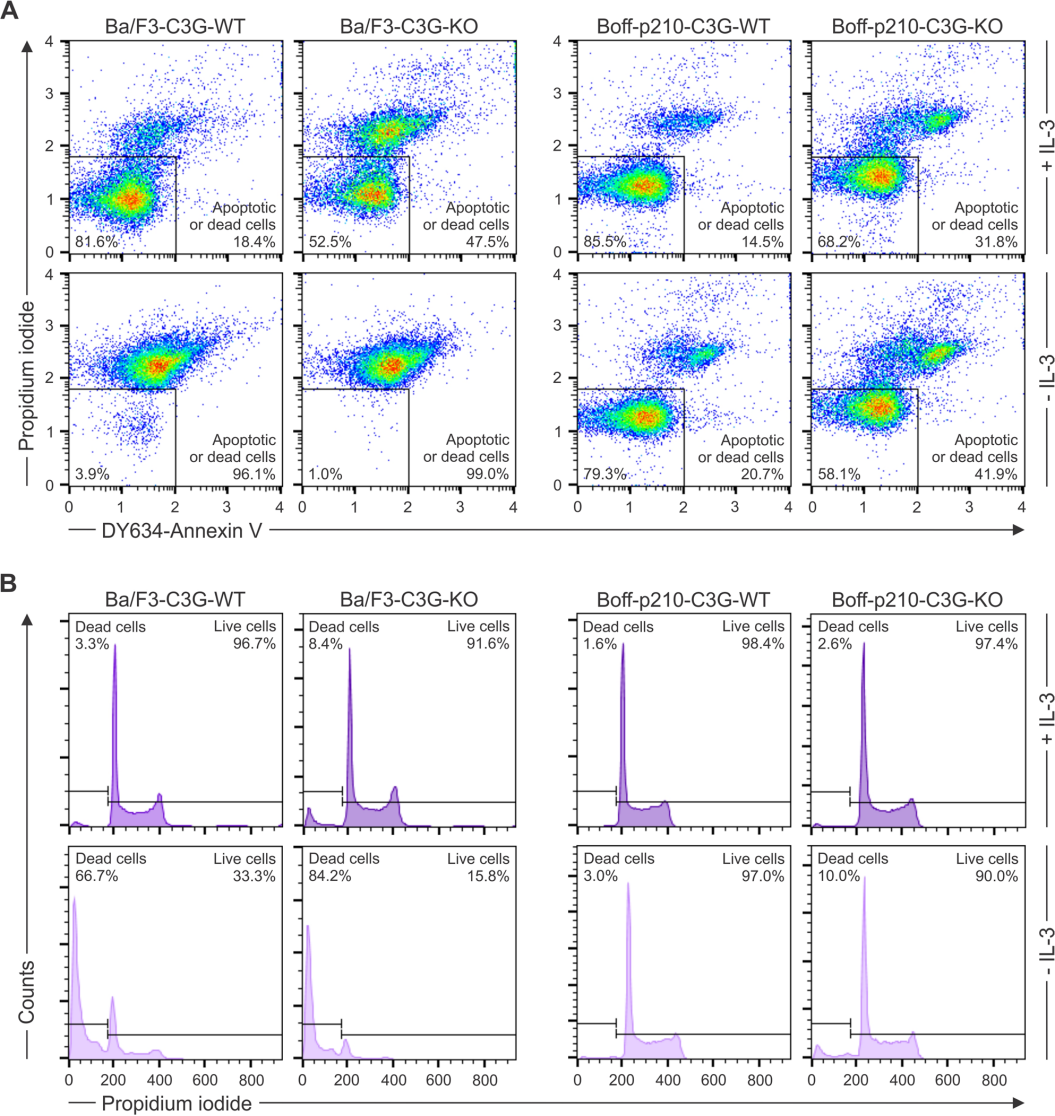
Inactivation of *Rapgef1* expression affects the survival of Ba/F3 and Boff-p210 cells. **A** Apoptosis analysis by Annexin V/propidium iodide (PI) labeling of Ba/F3 (left panels) and Boff-p210 (right panels) cells. Cells were harvested 72 h post-electroporation, by which point only late apoptotic events were detectable. The upper panels show cells cultured in the presence of IL-3 (+ IL-3), while the lower panels show cells cultured without IL-3 (- IL-3). Graph axes are in logarithmic scale, with the numbers corresponding to the exponent of 10. **B** Cell cycle phase distribution analysis by DNA content measurement. Ba/F3 (left panels) and Boff-p210 (right panels) cells were cultured in the presence (upper panels) or absence (lower panels) of IL-3. Cells were permeabilized and stained with PI. The percentage of live and dead cells is indicated

Previous studies from our group have shown that two missense mutations (Y554H and M555K), identified in non-Hodgkin lymphomas, lead to constitutive activation of C3G [38, 47]. To investigate the effects of these and other C3G mutants in B cells, we attempted to knock-out the C3G gene in the pro-B-cell lines Ba/F3 and Boff-p210 (IL-3-independent BCR::ABL1 p210-expressing Ba/F3) using CRISPR/Cas9 technology. *Rapgef1* inactivation (C3G-KO) increased cell death in both cell lines, compared to C3G-WT cells. In addition, Boff-p210-C3G-KO cells showed a higher dependence on IL-3 than Boff-p210-C3G-WT cells, as evidenced by an increase in apoptotic cells upon IL-3 withdrawal (from 31.8% to 41.9%) (Fig. 1A). This was accompanied by an approximate 7.5% point increase in DNA fragmentation (Fig. 1B). These findings suggest that C3G plays a critical role in cell survival, and support previous evidence indicating that C3G acts as an intermediary in the oncogenic effects of BCR::ABL1 [14, 48, 49].

We then attempted to isolate single-cell clones with C3G deletion from the analyzed cell pools. After culturing 384 clones, most did not grow. Among the survivors (52 Ba/F3 and 17 Boff-p210 clones), none were homozygous for the deletion. These results suggest that C3G is essential for B-cell growth.

### Successful generation of a C3G-Y564H knock-in cell line in A20 B-lymphoma cells

**Fig. 2:**
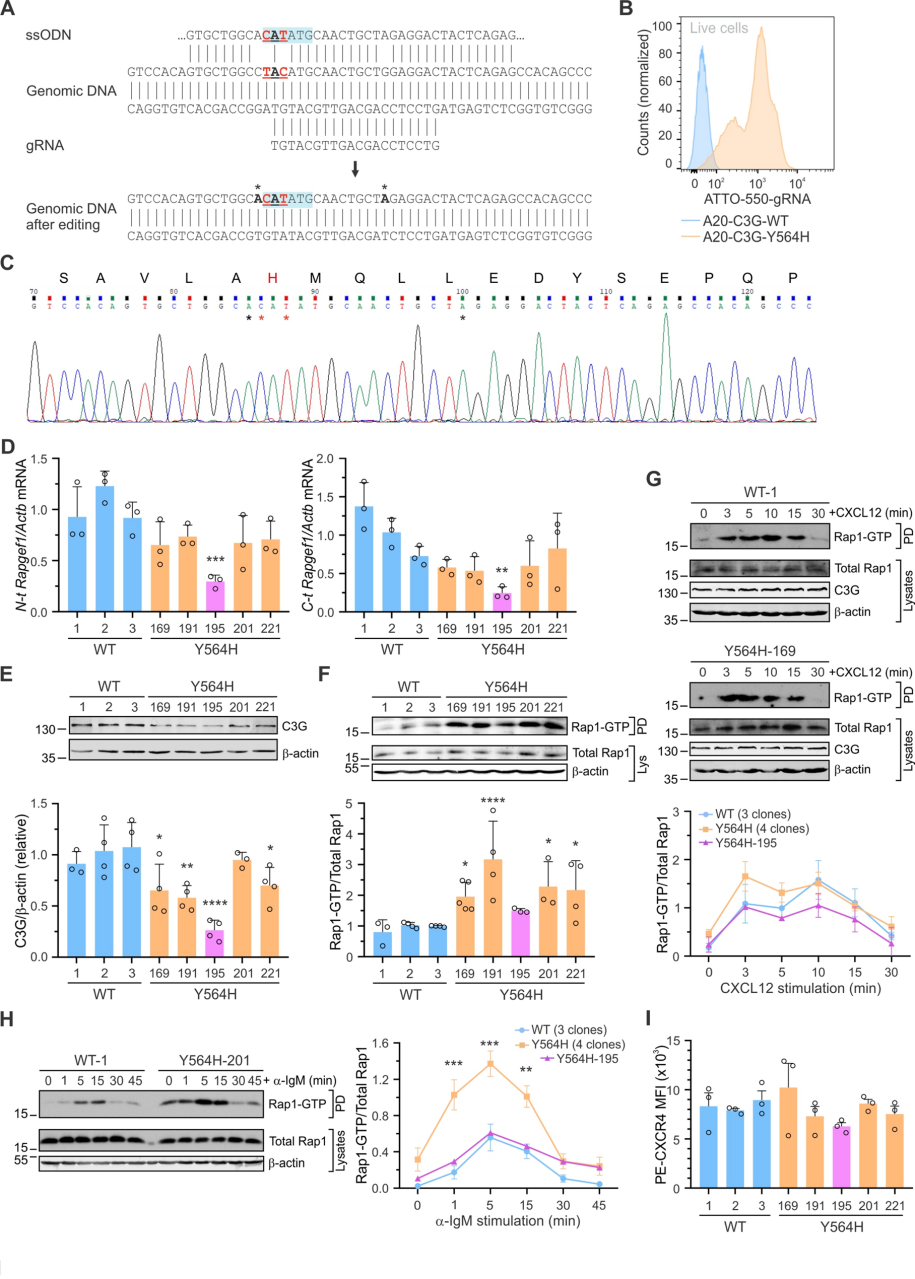
C3G-Y564H knock-in A20 cells show increased Rap1-GTP levels. **A** Upper: scheme of the gRNA and ssODN used to generate the C3G-Y564H mutant in A20 cells via CRISPR/Cas9. The two base pairs to be mutated to introduce the Y564H mutation are underlined in red. Asterisks indicate single PAM-silent mutations. Lower: predicted modification of the genomic DNA in the *Rapgef1* gene region, with the engineered NdeI restriction site shaded in blue. **B** Single-cell separation of clones derived from the cell pools. A20-C3G-WT: control cells electroporated with Cas9 protein alone, A20-C3G-Y564H: cells electroporated with the ribonucleoprotein targeting *Rapgef1*. **C** Sanger sequencing of one A20-C3G-Y564H clone. The protein sequence shows the HDR-generated H564 residue in red. Asterisks mark the mutated base pairs; red indicates changes in the 564 codon and black PAM-silent mutations. **D** RT-qPCR analysis (mean ± SD) of WT or mutated cells, using oligonucleotides targeting the *Rapgef1* N- (left panel) or C-terminal (right panel) regions (*n* = 3). Data were relativized to *Actb* expression. **E** C3G protein levels in A20-C3G-Y564H and A20-C3G-WT clones using anti-C3G (G-4) antibody. Upper panel: representative experiment; lower panel: mean ± SD of β-actin-relativized C3G levels (*n* = 3). **F** Steady-state levels of Rap1-GTP in A20 cells. Upper panel: representative experiment. Lower panel: mean ± SD of Rap1-GTP/Total Rap1 quantification (*n* = 3–5). **G**,** H** A20 cells were stimulated with (**G**) 100 ng/mL CXCL12 or (**H)** 10 µg/mL anti-IgM. Blots of representative pull-downs of active Rap1 and endogenous Rap1, C3G, and β-actin levels. Graphs show the quantification of Rap1 activation over time, relativized to total Rap1 expression (mean ± SEM), from grouped data, using three different A20-C3G-WT and four A20-C3G-Y564H clones. Results for the A20-C3G-Y564H-195 clone are shown separately. At least one experiment was performed for each clone. **I** CXCR4 surface expression (mean ± SEM, MFI) was assessed by flow cytometry using a PE-conjugated anti-mouse CXCR4 antibody. *p < 0.05, **p < 0.01, ***p < 0.001, ****p < 0.0001

Due to the challenges in generating C3G-KO clones in pro-B cells, we aimed to directly modify the endogenous C3G in B-cell lymphoma cells. To achieve this, we introduced the activating Y564H point mutation into the *Rapgef1* gene [38] in the A20 mouse B-cell lymphoma line using the CRISPR/Cas9 gene editing system with HDR. Therefore, the Y564H mutation was incorporated using a ssODN, homologous to the gDNA target region (Fig. 2A). The ssODN also included a NdeI restriction site and silent mutations in two additional PAM sequences (5’-NGG) to minimize excess Cas9 endonuclease activity after recombination. Single ATTO-550 positive cell clones were sorted by FACS (Fig. 2B) and expanded. The clones that survived (252) were genotyped using NdeI restriction fragment length polymorphism (RFLP) analysis. The sequences of the 54 apparently positive clones carrying the C3G-Y564H mutation were subsequently analyzed by Sanger sequencing (Fig. 2C). Five different knock-in clones were selected, named A20-C3G-Y564H-169, −191, −195, −201, and − 221. In parallel, we expanded 3 non-edited A20 clones (transfected only with the Cas9 enzyme), designated as A20-C3G-WT-1, −2, and − 3, which were used as controls.

### A20-C3G-Y564H cells exhibit increased Rap1 activation

First, we analyzed the expression of the *Rapgef1* gene in the A20-C3G-Y564H clones using RT-qPCR with two sets of primers (Fig. 2D) and confirmed C3G protein expression in the edited cells by western blot (Fig. 2E). Overall, both mRNA expression and protein levels were slightly reduced in the A20-C3G-Y564H clones compared to controls; however, significantly reduced C3G expression was observed only in the A20-C3G-Y564H-195 cells.

Next, we assessed the levels of active Rap1 in A20 cells expressing either C3G-WT or C3G-Y564H variants using pull-down assays with the GST-RalGDS-RBD fusion protein as bait. Under steady-state conditions, the C3G-Y564H mutation led to a basal increase in Rap1-GTP levels in A20 cells compared to the C3G-WT form (Fig. 2F), consistent with observations in transiently transfected HEK-293T and Ba/F3 cells [38]. The A20-C3G-Y564H-195 clone showed only a slight increase in Rap1-GTP levels, likely attributable to its reduced C3G expression (Fig. 2D, E).

Afterwards, we explored whether C3G-Y564H could enhance Rap1 GTPase activation in A20 cells upon B-cell activation with various stimuli. Activation of CXCR4 (by CXCL12) or the BCR (by anti-IgM) triggered transient Rap1 activation in the A20 clones (Fig. 2G, H). BCR stimulation led to a significant increase in Rap1-GTP levels in the C3G-Y564H mutant clones compared to the C3G-WT control clones (Fig. 2H), except for the A20-C3G-Y564H-195 clone. Increased Rap1-GTP levels were also observed in A20 cells ectopically expressing the human C3G-Y554H-EGFP construct (Additional file 1: Fig. **S2**). The effect of the C3G-Y564H mutation was less pronounced upon CXCL12 stimulation, however the A20-C3G-Y564H-195 clone again displayed substantially, though no significantly, lower Rap1-GTP levels compared to the other A20-C3G-Y564H clones (Fig. 2G). In addition to reduced C3G expression, this may be partly attributed to the slightly decreased surface expression of CXCR4 observed in A20-C3G-Y564H-195 cells (Fig. 2I).

In summary, the C3G-Y564H mutation enhanced both basal and stimuli-induced Rap1 activation (mainly through BCR) in A20 cells, except in the − 195 clone, likely due to its reduced C3G expression. As C3G deregulation and expression levels appear to play distinct roles in A20 cells, we will differentiate between the A20-C3G-Y564H-195 clone and the other mutant clones in subsequent analyses.

### Hyperactivation of the C3G-Rap1 pathway impairs proliferation and increases apoptosis in A20 cells

**Fig. 3:**
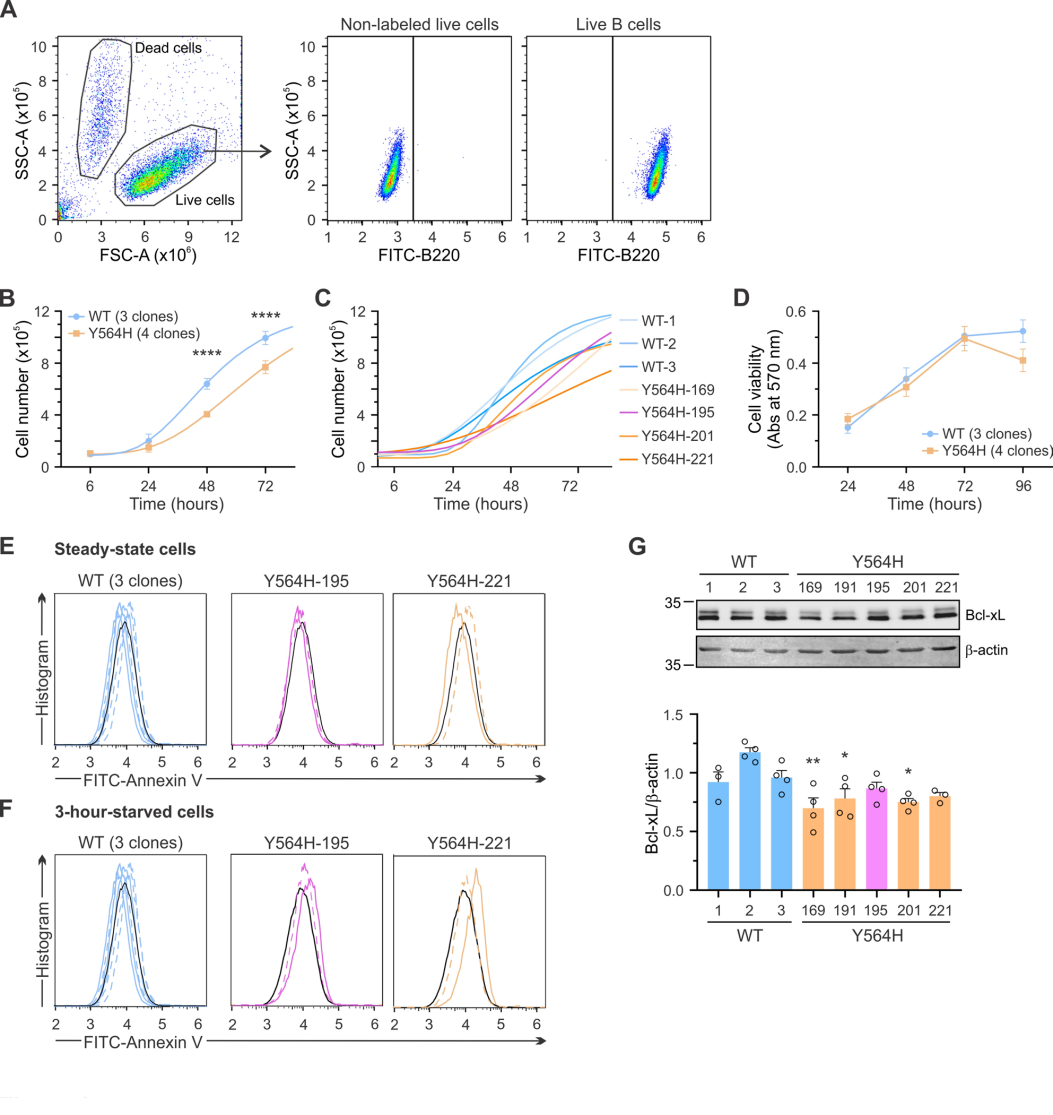
A20-C3G-Y564H cells show lower proliferation and increased apoptosis, without changes in cell viability. **A-C** Live cells quantified by flow cytometry at the indicated time points. Cells were harvested and labeled with a FITC-conjugated anti-mouse CD45R/B220 antibody. **A** Representative flow cytometry analysis. Left panel: FSC vs. SSC dot plot distinguishing live from dead cells. Middle panel: FITC-B220 vs. SSC dot plot, used as a control for antibody labeling after gating live cells. Right panel: quantification of B220-positive live cells, used for proliferation analysis. **B** Time course of A20 cell growth. The graph shows the mean ± SEM of the grouped number of live, B220-positive, cells from three A20-C3G-WT and four A20-C3G-Y564H clones. Lines represent four-parameter Gompertz growth models fitted to the experimental data. ****p < 0.0001. **C** Growth models were independently fitted to the proliferation data of individual cell clones. Two independent experiments were conducted for each clone, with experimental triplicates per clone. **D** A fixed number of cells (~ 2 × 10⁴) was seeded, and cell viability was assessed over 96 h using the MTT assay, measuring absorbance at 570 nm. The graph represents the mean ± SEM of grouped data from three A20-C3G-WT and four A20-C3G-Y564H clones. Two independent experiments were performed for each clone, with experimental triplicates per clone and time point. **E**,** F** Apoptosis analysis of A20-C3G-Y564H and A20-C3G-WT cells by flow cytometry using FITC-Annexin V (**E**) under steady-state conditions and (**F**) after starvation in medium containing 0.5% FBS for 3 h. The average apoptosis of A20-C3G-WT cells is depicted by the solid black line. **G** Analysis of Bcl-xL expression in A20-C3G-Y564H and A20-C3G-WT cells by western blot. Upper panel: representative experiment. Lower panel: Quantification (mean ± SEM) of Bcl-xL levels relative to β-actin from four independent experiments. *p < 0.05, **p < 0.01

One of the key hallmarks of cancer cells is their ability to sustain proliferative signaling, leading to continuous and uncontrolled cell proliferation [50]. Therefore, we aimed to determine whether the C3G-Y564H mutation could affect the proliferative ability of A20 cells. Flow cytometry using a B-cell-specific conjugated antibody (FITC-B220) was employed to quantify live cell numbers at different time points after cell seeding (Fig. 3A). A20-C3G-Y564H cells exhibited a significantly lower cell proliferation rate compared to A20-C3G-WT cells (Fig. 3B). A general growth difference was observed between wild-type and mutant clones, based on the analysis of four mutant and three wild-type clones (Fig. 3C). Similar results were obtained by live-cell imaging microscopy (Additional file 1: Fig. S1A-C), except for the A20-C3G-Y564H-195 clone, which displayed a growth pattern closer to those of A20-C3G-WT cells, in line with its lower C3G expression. No significant differences in cell viability, as measured by MTT metabolism, were observed between the mutant and control clones (Fig. 3D). These results are consistent with a tumor suppressor role for C3G in B-cell lymphoma, as described in other tumorigenic models [4–7, 12, 16, 17].

C3G can exert a pro- or anti-apoptotic effect depending on the cellular context [48, 51–53]; therefore, we next investigated whether the altered cell growth was related to increased apoptosis due to C3G deregulation. Apoptosis was measured in A20-C3G-WT-1, −2, −3 clones, and A20-C3G-Y564H-195, −221 clones, under both steady-state conditions and stress induced by serum deprivation for 3 h. No differences were observed under resting conditions (Fig. 3E). However, serum-starved A20-C3G-Y564H cells, particularly the 221 clone, exhibited slightly increased apoptosis compared to A20-C3G-WT cells (Fig. 3F). Consistently, A20-C3G-Y564H cells showed a trend toward reduced levels of the anti-apoptotic protein Bcl-xL compared to control cells (Fig. 3G). This increased apoptosis may contribute to the suppressive effect of the C3G-Y564H mutation on A20 cell growth.

### A20-C3G-Y564H cells present reduced expression of c-Raf and decreased MEK1/2 and ERK1/2 activation

**Fig. 4:**
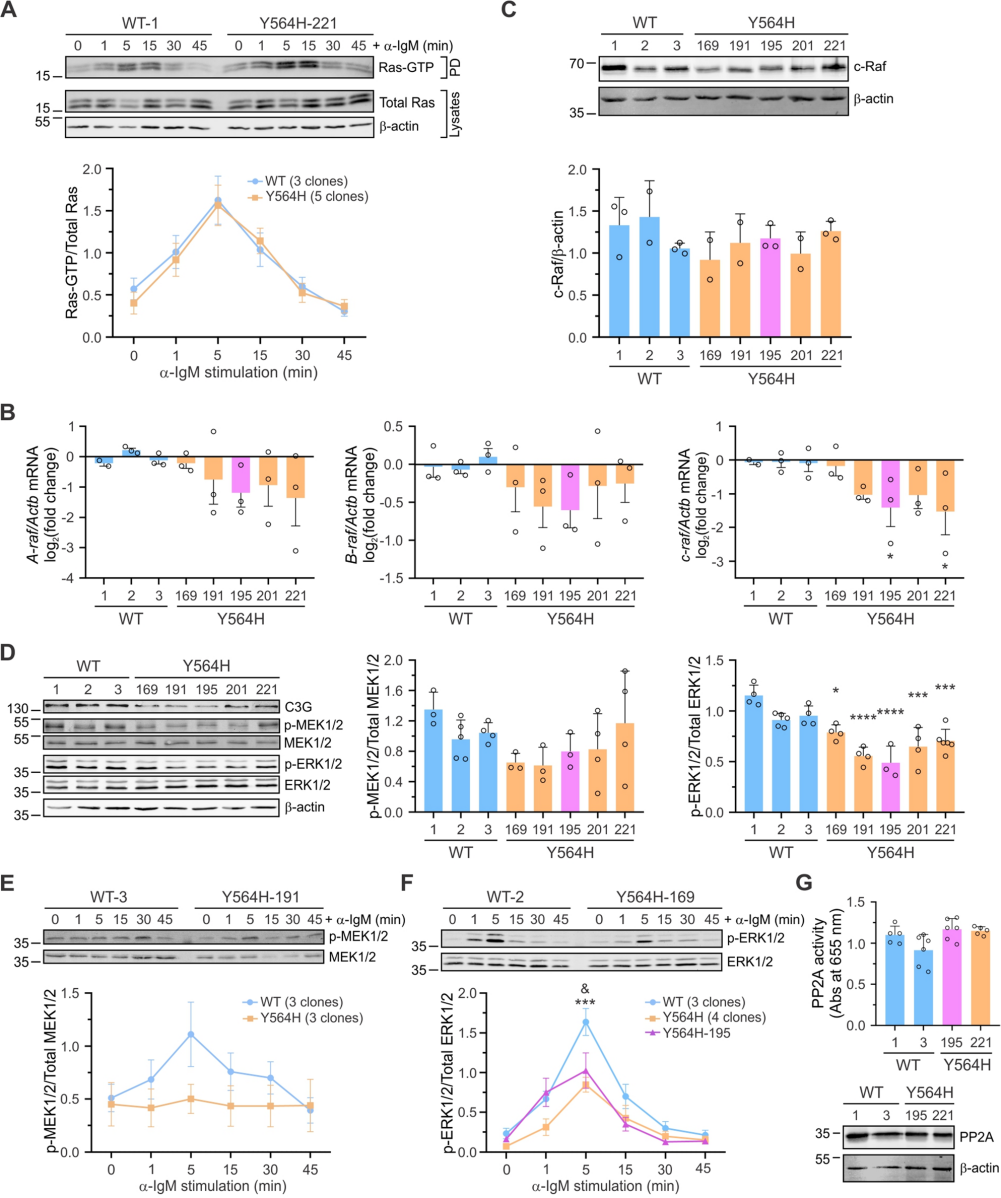
A20-C3G-Y564H cells show reduced c-Raf, phospho-MEK1/2, and phospho-ERK1/2 levels. **A** Time course of Ras-GTP levels in cells stimulated with 10 µg/mL anti-IgM. Upper panel: representative pull-down experiment. Lower panel: Ras-GTP/Total Ras ratio (mean ± SEM) from grouped data, using three A20-C3G-WT and five A20-C3G-Y564H clones. **B** RT-qPCR analysis of *A-raf*,* B-raf*, and *c-raf* mRNA expression, relative to *Actb* levels. Results are shown as log_2_(fold change) and are normalized to values in A20-C3G-WT cells. **C** Representative western blot and mean ± SD of c-Raf levels relative to β-actin, normalized to the average levels in A20-C3G-WT cells, from at least two independent experiments with each clone. **D** MEK1/2 and ERK1/2 activation in resting cells. Values were normalized to the total MEK1/2 or ERK1/2 protein levels. C3G and β-actin served as additional controls. A representative experiment is shown. Bar charts show the mean ± SD of p-MEK1/2 to total MEK1/2 (left) and p-ERK1/2 to total ERK1/2 (right) ratios from at least three independent experiments. Values of A20-C3G-Y564H clones were compared to the average levels in A20-C3G-WT cells. **E**,** F** A20 clones were stimulated with 10 µg/mL anti-IgM, and the ratios of (**E**) p-MEK1/2/Total MEK1/2 and (**F**) p-ERK1/2/Total ERK1/2 quantified (mean ± SD). Upper: representative western blots. Lower: mean ± SEM of p-MEK1/2 and p-ERK1/2 levels, relative to total MEK1/2 or ERK1/2 levels (grouped data), using three A20-C3G-WT and three A20-C3G-Y564H clones. Results for the A20-C3G-Y564H-195 clone are presented separately (*n* = 3). **G** PP2A activity (mean ± SD). Two independent experiments were performed with each indicated clone, using experimental triplicates per clone. The lower panel shows a representative western blot anti-PP2A of the cell lysates used for immunoprecipitation. *p < 0.05, ***p < 0.001, ****p < 0.0001 when comparing A20-C3G-WT with A20-C3G-Y564H cells. &*p* < 0.05 when comparing A20-C3G-WT cells with the A20-C3G-Y564H-195 clone. At least one experiment of each type was performed with every clone

The Ras-ERK1/2 signaling pathway is a critical regulator of cell proliferation and apoptosis [54]; therefore, we investigated whether the C3G-Y564H mutation could impact this pathway. We first analyzed Ras activation in response to BCR stimulation and observed no differences in Ras-GTP levels between A20-C3G-Y564H and A20-C3G-WT cells (Fig. 4A). Next, we analyzed Raf levels as downstream effectors of Ras and found lower mRNA expression levels of all three Raf isoforms—*A-raf*, *B-raf*, and *c-raf*—in A20-C3G-Y564H clones compared to control clones (Fig. 4B). The most pronounced effect was observed in the c-Raf isoform, the primary Raf protein variant involved in B-cell signaling upon BCR stimulation [55]. Notably, c-Raf protein levels also exhibited a decreasing trend in A20-C3G-Y564H cells (Fig. 4C). Phosphorylation of MEK1/2 and ERK1/2 was reduced in the mutant clones, both at steady-state (Fig. 4D) and upon BCR stimulation (Fig. 4E, F), with the reduction being more pronounced in phospho-ERK1/2, consistent with signal amplification. It is worth mentioning that the A20-C3G-Y564H-195 clone exhibited p-ERK1/2 levels intermediate between those of control cells and the other mutant clones when stimulated with anti-IgM (Fig. 4F), in line with its lower C3G expression and Rap1 activation (Fig. 2E-H). In agreement with these results, we detected in A20-C3G-Y564H cells differential expression of several genes regulated by ERKs [56], such as *Phlda1*, *Dusp4*, *Dusp6*, *Spry2*, *Etv4*, and *Etv5* (Additional file 1: Fig. S3A-F). It has been reported that C3G can reduce phospho-ERK1/2 levels through the activation of PP2A phosphatases [18]. However, no differences in PP2A activation levels were observed between mutant and control cells (Fig. 4G).

Collectively, these data indicate that the C3G-Y564H mutation affects A20 cell proliferation by interfering with the Ras-ERK1/2 pathway, possibly through competition between Ras and Rap1 for c-Raf binding, as previously described [15]. Additionally, the reduced *Raf* mRNA expression observed in the mutant clones suggests a role for C3G in transcriptional regulation.

### Cell adhesion is altered in A20-C3G-Y564H cells

**Fig. 5:**
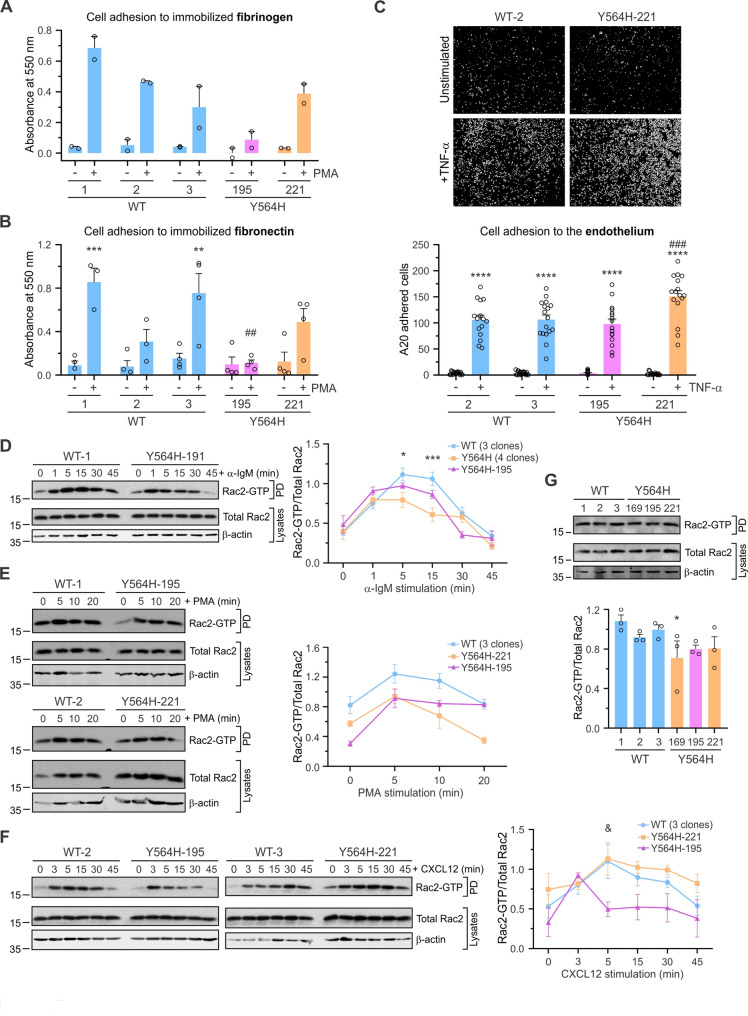
Altered adhesion in A20-C3G-Y564H cells correlates with defective Rac2 activation. **A**,** B** Adhesion of cells, treated or not with 20 nM PMA, to plates coated with (**A**) fibrinogen or (**B**) fibronectin. The bar charts represent the mean ± SEM of absorbance measure at 550 nm. At least two independent experiments were performed with each clone, using experimental triplicates per clone. ##*p* < 0.01 versus PMA-stimulated A20-C3G-WT cells. **p < 0.01, ***p < 0.001 versus the corresponding basal state level of each clone. **C** A20 cell adhesion to a confluent HUVEC monolayer, either unstimulated or treated with 10 ng/mL TNF-α overnight. Adhesion was quantified after 60 min at 37 °C. At least three independent experiments were performed with each clone (6 wells/condition in each experiment). Upper: representative binary images obtained after applying the Labkit plugin; lower: the bar charts represent the number of adhered cells on HUVECs (mean ± SEM) relativized to the adhered A20-C3G-WT cells without TNF-α. ###*p* < 0.001 versus PMA-stimulated A20-C3G-WT cells. ****p < 0.0001 versus the corresponding basal state level of each clone. **D-G** Analysis and quantification of active Rac2 (Rac2-GTP) after stimulation with (**D**) 10 µg/mL anti-IgM, (**E**) 10 ng/mL PMA, (**F**) 100 ng/mL CXCL12, or (**G**) at steady-state. Rac2 and β-actin expression in cell lysates were analyzed as controls. Representative western blots are shown in the left panels. The graphs show the mean ± SEM of the Rac2-GTP/Total Rac2 ratio of (**D-F**) grouped data from three A20-C3G-WT and four different A20-C3G-Y564H clones ((**D**) five or (**E**,** F**) three independent experiments, with at least one experiment performed for each clone). Results for the A20-C3G-Y564H-195 clone presented separately. **G** Data were normalized to the average Rac2 protein levels in A20-C3G-WT cells for each experiment. *p < 0.05, ***p < 0.001 when comparing A20-C3G-WT with A20-C3G-Y564H cells. &*p* < 0.05 when comparing A20-C3G-WT with the A20-C3G-Y564H-195 clone

One of the primary functions of the C3G-Rap1 pathway is the regulation of cell adhesion and migration. Indeed, hyperactivation of this pathway has been associated with hematologic malignancies due to aberrant integrin-mediated cell adhesion [13, 49]. This led us to investigate whether the C3G-Y564H mutation could affect the adhesive capacity of A20 cells. We first assessed the ability of A20-C3G-Y564H cells, with or without PMA stimulation, to bind soluble fibrinogen, which interacts with the integrin αXβ2 present on B cells [57, 58]. Additional file 1: Fig. S4A shows that A20-C3G-Y564H cell clones 195 and 221 bound fibrinogen to a lesser extent than A20-C3G-WT cells, and that PMA stimulation did not significantly affect this interaction. Next, we analyzed the adhesion of the clones to immobilized fibrinogen and fibronectin. Under resting conditions, none of the clones adhered noticeably to fibrinogen- or fibronectin-coated plates (Fig. 5A, B). Upon PMA stimulation, cell adhesion was significantly increased in A20-C3G-WT cells. However, only the A20-C3G-Y564H-221 clone, and not the A20-C3G-Y564H-195 clone, responded to PMA-induced adhesion, albeit this response was generally lower than that of the control clones, particularly on fibronectin (Fig. 5A, B). This suggests that C3G may influence B-cell adhesion to ECM components independently of Rap1, with C3G expression levels—rather than its GEF activity—playing the primary role. This hypothesis is supported by the observation that Rap1 activation was increased in both A20-C3G-Y564H clones upon PMA stimulation, compared to control clones (Additional file 1: Fig. S4B). In contrast, A20-C3G-Y564H-221 cells exhibited increased adhesion to TNF-α-stimulated HUVEC monolayers compared to control cells (Fig. 5C), suggesting a role for the C3G-Rap1 axis in transendothelial migration—a function of Rap1 that has been previously reported [29]. The A20-C3G-Y564H-195 clone showed lower adhesion to HUVECs than the A20-C3G-Y564H-221 clone (Fig. 5C), likely linked to its reduced Rap1-GTP levels (Fig. 2F). No alterations were observed in the surface expression of the integrins LFA-1 (αLβ2), which binds ICAM on HUVECs, or VLA-4 (α4β1), which binds fibronectin [57], in A20-C3G-Y564H cells. An exception was the 195 clone, which exhibited increased VLA-4 levels, likely as a compensatory mechanism for its impaired adhesion (Additional file 1: Fig. S4C). Similarly, no differences in Talin-1 expression, a key Rap1-mediated regulator of integrins [59], were observed in A20-C3G-Y564H cells compared to A20-C3G-WT cells (Additional file 1: Fig. S4D).

Collectively, these results indicate that B-cell adhesion to ECM components is highly sensitive to C3G levels, likely through interactions with other proteins, such as focal adhesion (FA) proteins via its proline-rich region [49], whereas adhesion to endothelial cells appears to rely on C3G-mediated Rap1 activation.

### C3G deregulation leads to reduced Rac2 activity

Both BCR and CXCR4 induce B-cell adhesion through pathways involving Rho GTPases [60]. In particular, Rac2-deficient cells exhibit adhesion defects linked to reduced Rap1 activation [37]. Therefore, we investigated whether C3G hyperactivation influences the activation of Rho GTPases in our B-cell model. Pull-down assays revealed that the C3G-Y564H mutation did not significantly impact Rac1 (Additional file 1: Fig. S4E) or Cdc42 activation (Additional file 1: Fig. S4F) following BCR stimulation. In contrast, A20-C3G-Y564H clones exhibited a significant reduction in Rac2-GTP levels compared to A20-C3G-WT clones, particularly at peak of the time course (Fig. 5D). An exception was the A20-C3G-Y564H-195 clone, which displayed intermediate Rac2-GTP levels, falling between those of the wild-type controls and the other mutant clones. A similar intermediate level of activation was also observed following PMA stimulation (Fig. 5E). CXCL12 also activates Rac2 in A20 cells, with no significant differences between mutant and control clones. However, the A20-C3G-Y564H-195 clone exhibited reduced Rac2-GTP levels compared to WT clones (Fig. 5F). Only minor differences in Rac2 activation were observed between mutant and control clones under steady-state conditions (Fig. 5G).

These results suggest that C3G negatively regulates Rac2 activity—likely in a Rap1-independent manner—in response to different stimuli, which may contribute to the decreased adhesion of C3G mutant A20 clones, particularly the C3G-Y564H-195 clone, on certain ECM substrates.

### The C3G-Y564H mutation leads to enhanced migration and invasion of A20 cells

**Fig. 6:**
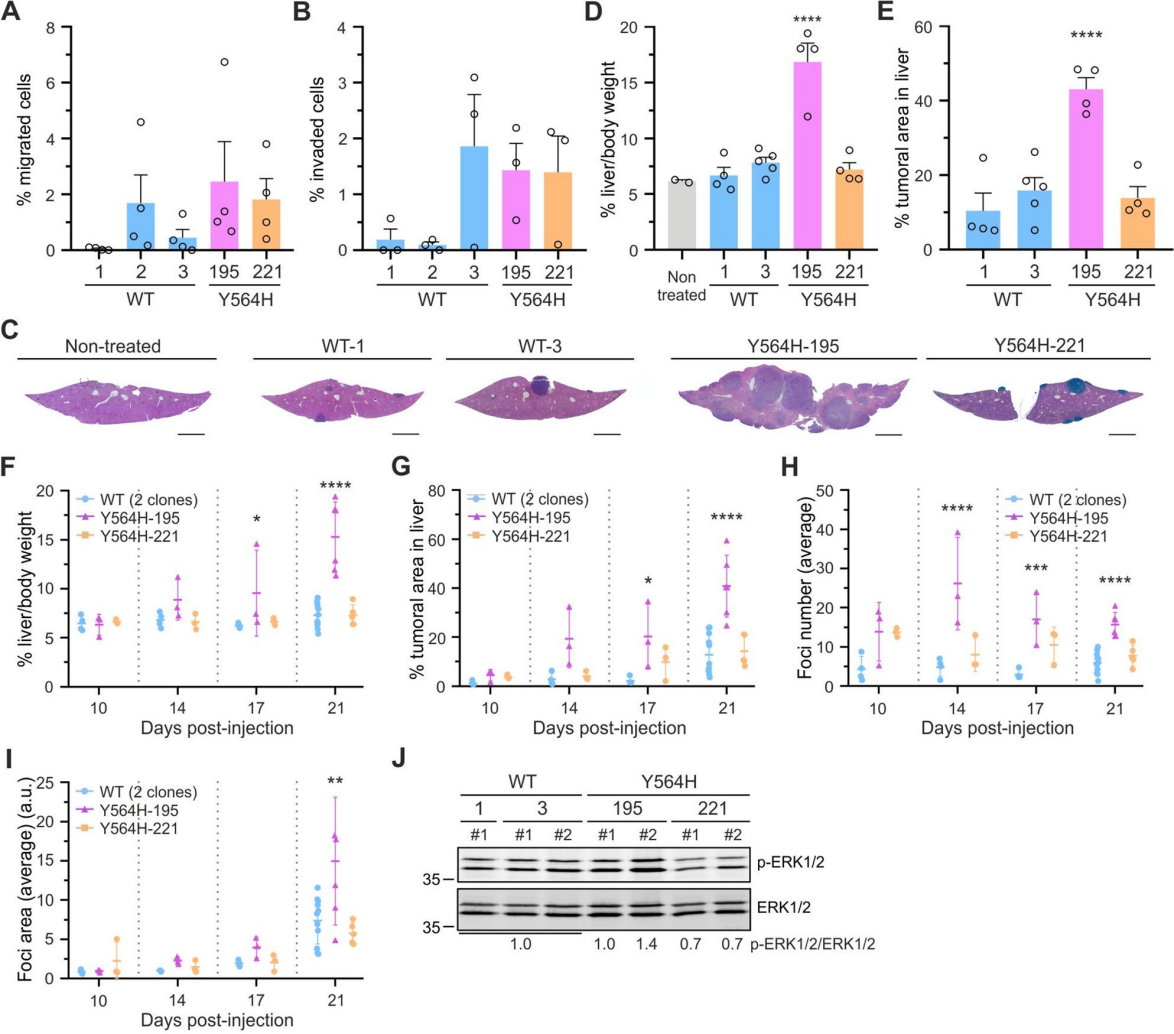
C3G hyperactivation enhances the metastatic potential of A20 cells. **A** Bar charts of the percentage of migrated cells in Transwell assays (mean ± SEM) relative to the initial seeded cells. Four independent experiments were performed. **B** Invasion of cell in Matrigel-coated Transwell assays (mean ± SEM) relative to the initial seeding. Three independent experiments were performed. **C** Representative liver sections from mice injected with A20-C3G-WT (clones 1 and 3) or A20-C3G-Y564H cells (clones 195 and 221), 21 days post-injection. A liver section from a non-treated BALB/c mouse serves as a control. Histopathological analysis was performed using hematoxylin and eosin staining. Scale bar: 0.2 cm. **D**,** E** Percentage of (**D**) liver weight to total body weight (mean ± SEM) and (**E**) tumor cell area as a percentage of the total liver section area (mean ± SEM). At least four mice injected with each cell clone were analyzed in four independent experiments. **F** Liver weight as a percentage of total body weight. **G-I** Graphs displaying the mean ± SEM of the following parameters (each data point represents one liver, with all foci analyzed from four slices taken from the same regions): (**G**) average tumor cell area as a percentage of the total liver section area, (**H**) average foci number, and (**I**) average foci area. **J** Representative western blots showing the ratios of p-ERK1/2/Total ERK1/2 in lysates from liver metastases of the indicated clones, 21 days post-injection. Ratios are normalized to the mean value of the three WT samples. Numbers preceded by an # are used to designate independent mice. *p < 0.05, **p < 0.01, ***p < 0.001, ****p < 0.0001 versus data from mice injected with A20-C3G-WT cells. a.u.: arbitrary units

Rac GTPases play a key role in regulating cytoskeletal structures and, consequently, in cell migration and invasion [61]. Similarly, Rap1 is essential for A20 cells to undergo transendothelial migration [29], and its reduced activity in B cells leads to impaired migration toward CXCL12 [28]. Therefore, we assessed migration over a 48-hour period using Transwell assays. Overall, A20-C3G-Y564H cells exhibited a greater migratory capacity than A20-C3G-WT cells (Fig. 6A), although the differences were not statistically significant. Similar results were obtained in invasion assays, using Transwell inserts coated with Matrigel (Fig. 6B). These results correlate with the decreased adhesion to the ECM observed in the mutant clones (Fig. 5A, B).

### The C3G-Y564H mutation increases metastasis but reduces the size of A20-induced tumor foci in vivo

The in vitro studies above suggest an antitumoral phenotype for the A20-C3G-Y564H mutant clones, except for their tendency toward increased endothelial adhesion, migration, and invasion. To further investigate the role of the Y564H mutation in A20 cell biology, we analyzed its effect on the metastatic properties of A20 cells in BALB/c mice. This syngeneic model of murine B-cell lymphoma has been described to closely mimic the gross histopathologic and cytomorphologic characteristics of human B-cell lymphomas [62]. A20-C3G-Y564H-195, −221, and two A20-C3G-WT clones were intravenously injected into BALB/c mice, and metastases in the liver and spleen were analyzed up to 21 days. Only A20-C3G-Y564H-195-injected mice exhibited significant hepatomegaly and a tendency toward splenomegaly (Fig. 6C, D, F and Additional file 1: Fig. S5A-C), accompanied by a significantly larger metastatic area in the liver (Fig. 6C, E, G). Additionally, A20-C3G-Y564H-195 cells showed an increased metastatic area in the spleen (Additional file 1: Fig. S5D, E). A closer examination revealed that A20-C3G-Y564H-221 mutant cells also induced a greater number of tumor foci in the liver than the A20-C3G-WT cells (Fig. 6H). However, these foci were smaller than those induced by control cells 21 days post-injection (Fig. 6I), suggesting reduced tumor growth. Consistently, similar results were observed in the livers of mice injected with A20 cells ectopically expressing the C3G-Y554H mutant (Additional file 1: Fig. S5F-J). This finding aligns with the antiproliferative properties of the A20-C3G-Y564H clones and their increased invasive potential. In contrast, the A20-C3G-Y564H-195 clone exhibited a more aggressive phenotype, as evidenced by the increased number and larger size of tumor foci in liver (Fig. 6H, I), in line with the observed splenomegaly and expanded tumor area. This aggressive behavior may be attributed to the reduced C3G expression and basal Rap1 activation observed in this clone, which are associated with its increased cell proliferation compared to the other mutant clones (Additional file 1: Fig. S1A-C). This is further supported by the results in Fig. 6J, showing reactivation of p-ERK1/2 levels in A20-C3G-Y564H-195-induced liver metastasis.

Collectively, these data support a dual role for C3G in lymphoma, stemming from both altered activity and expression: hyperactivated C3G functions as a tumor suppressor by inhibiting cell proliferation and promoting apoptosis through Rap1-dependent inhibition of the Raf-ERK1/2 pathway, while concurrently reducing adhesion—likely via downregulation of Rac2 activation—thereby enhancing migration and invasion. In addition, constitutive C3G activation contributes to Rap1-mediated endothelial adhesion. However, the increased tumorigenic potential of the C3G-Y564H-195 clone is more likely attributable to its reduced C3G expression rather than catalytic deregulation.

### C3G hyperactivation and altered expression levels lead to changes in the gene expression profile of A20 cells

**Fig. 7:**
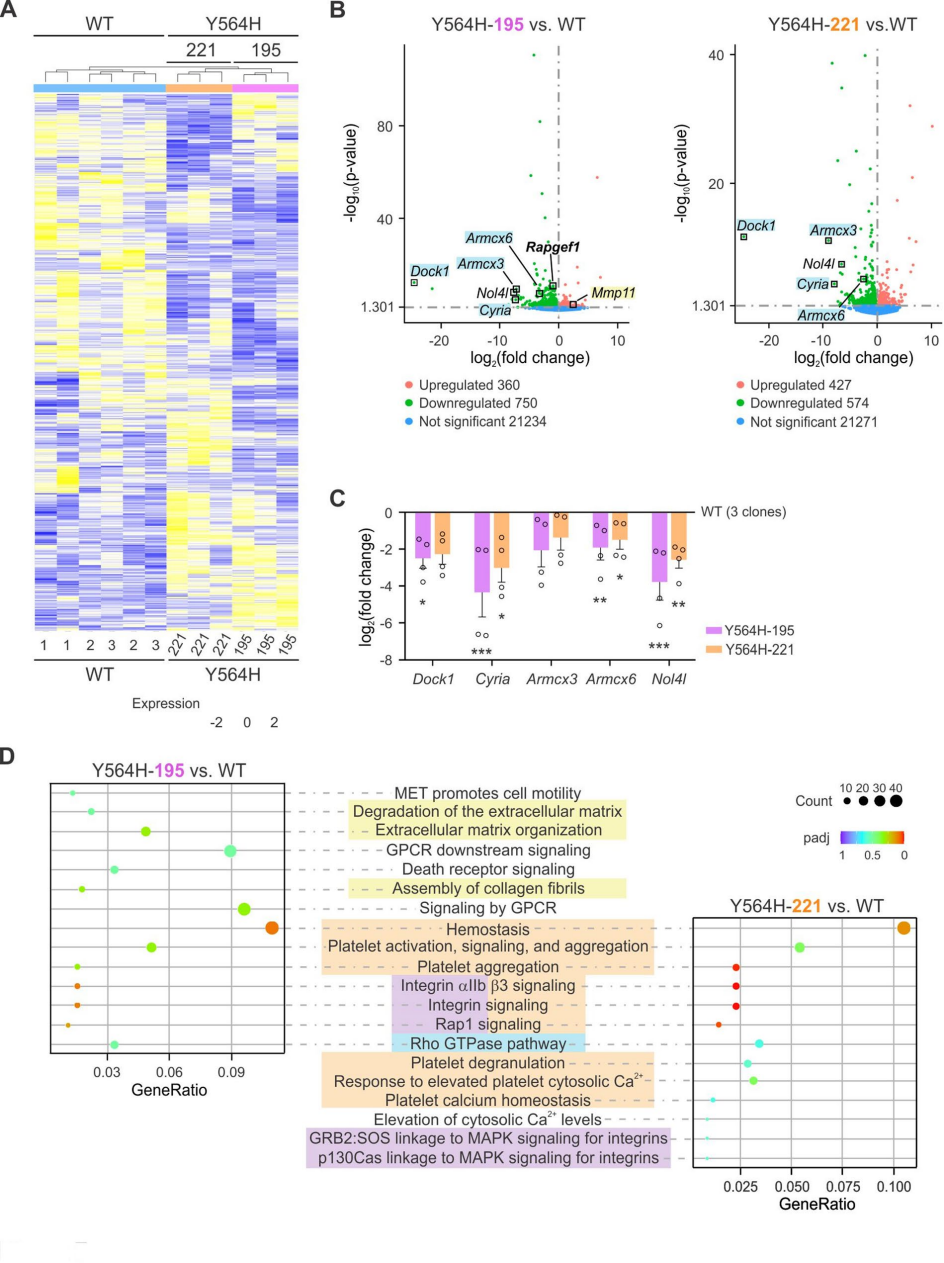
Transcriptomic analysis of A20 cells with catalytically deregulated C3G. **A** Heatmap of differentially expressed genes (DEGs) in A20-C3G-WT clones (−1, −2, and − 3; each in duplicate) and A20-C3G-Y564H mutant clones (−195 and − 221; each in triplicate). Hierarchical clustering was performed based on gene expression profiles, grouping genes and samples with similar patterns. Red indicates high expression levels and green indicates low expression levels. Sample names are shown at the bottom; clustering dendrograms for genes and samples are shown at the top and left sides. **B** Volcano plots representing DEGs for each mutant clone compared to pooled A20-C3G-WT samples. The DEGs of particular interest are highlighted. **C** mRNA expression levels *Dock1*, *Cyria*, *Armcx3*, *Armcx6* and *Nol4l* in A20-C3G-Y564H-195 and − 221 clones analyzed by RT-qPCR using specific oligonucleotides. Bar chart represent the mean ± SEM of the log_2_(fold change) from four independent experiments, relative to the averaged expression levels in A20-C3G-WT cells (clones 1, 2, and 3). *p < 0.05, **p < 0.01, ***p < 0.001. **D** Pathway enrichment analysis using the Reactome database. The most significantly enriched pathways for each mutant clone are shown. Shared pathways between the two mutant clones are aligned in the center, while clone-specific pathways appear on the upper (A20-C3G-Y564H-195 cells) or bottom (A20-C3G-Y564H-221) sides of the panel. Genes related to ECM degradation, platelet function, integrin function, and Rho GTPase signaling are highlighted with yellow, orange, light purple, and blue shading, respectively. Padj: adjusted p-value. Count: number of genes from the input list that map to a given pathway

To further investigate the molecular mechanism underlying the distinct behavior of the mutant clones, we performed RNA sequencing to obtain detailed transcriptome profiles for each cell type. Gene expression analyses were conducted using three independent A20-C3G-WT clones (pooled as a control) and the A20-C3G-Y564H-195 and − 221 clones. A hierarchical clustering heatmap, based on 2242 DEGs, revealed that all samples from the three different clone groups (WT, Y564H-195 and 221) clustered together. Notably, both mutant clones showed gene expression patterns that were nearly antagonistic to those of the WT cells, suggesting that the C3G-Y564H mutation exerts a strong epigenetic influence. Differences in the transcriptome profiles were also observed between the A20-C3G-Y564H-195 and − 221 clones (Fig. 7A), in line with the distinct functional behavior exhibited by the two clones.

Focusing on the DEGs in both mutant clones compared to A20-C3G-WT cells, we confirmed that *Rapgef1* expression was specifically downregulated in A20-C3G-Y564H-195 cells, but not in −221 clone (Fig. 7B), consistent with previous results (Fig. 2D, E).

In line with both in vitro and in vivo experiments pointing to a key role for Rac2 in our model, several genes involved in Rac2 signaling—*Dock1*, *Cyria*, *Armcx3*, and *Armcx6*—were consistently downregulated in both mutant clones (Fig. 7B). These changes were further validated by gene expression analysis using RT-qPCR (Fig. 7C), and by pathway enrichment analysis through the Reactome database, which identified significant enrichment of the Rho GTPase signaling pathway in both mutant clones (Fig. 7D). Furthermore, C3G deregulation caused by the Y564H mutation was associated with enrichment of multiple pathways related to platelet biology (Fig. 7D). These findings further support previous evidence highlighting the central role of the C3G–Rap1 axis in platelet function [22, 23, 36, 63–66].

Integrin signaling also emerged as one of the top enriched pathways, potentially explaining the increased adhesion of A20-C3G-Y564H-221 cells to endothelial cells (Fig. 5C), as well as their enhanced migration and invasion capacities in vitro (Fig. 6A, B).

Among the two mutant clones, A20-C3G-Y564H-195 exhibited more pronounced alterations in ECM-related processes (Fig. 7D), likely driven by dysregulation of matrix-remodeling genes such as *Mmp11*. Combined with its reduced adhesion to fibrinogen and fibronectin (Fig. 6C, E), this may explain its more invasive in vivo phenotype compared to the A20-C3G-Y564H-221 clone (Figs. 5A and B and 6B-E). It is also noteworthy that the expression of *Nol4l* (*C20orf112*), a gene associated with B-cell lymphoma and leukemia, was significantly reduced in both mutant clones (Fig. 7B, C). When fused to *Pax5*—a key regulator of B-cell development—the resulting Pax5-Nol4l fusion protein acts as a dominant-negative regulator of B-cell differentiation [67].

Altogether, the transcriptomic data confirm and support the biological insights inferred from the functional characterization of our B-cell model with catalytic dysregulation of C3G.

## Discussion

In this study, we demonstrate that the endogenously generated C3G activating mutation, Y564H, introduced via CRISPR/Cas9 in the murine B-cell line A20, suppressed proliferation and survival while enhancing endothelial adhesion, migration, and invasion, likely through Rap1-dependent mechanisms. Furthermore, C3G decreased cell adhesion to the ECM, presumably by modulating the Rac2 pathway, independently of Rap1. This bifunctional role of C3G—mediating both Rap1-dependent and -independent effects—has been extensively documented [16–18, 48, 53].

Previous in vitro studies using transfected HEK-293T and Ba/F3 cell lines demonstrated that the equivalent mutation in human C3G (Y554H) increases steady-state Rap1-GTP levels [38], consistent with the results observed in our knock-in A20-C3G-Y564H cells. This hyperactivation of the C3G-Rap1 pathway was associated with decreased cell proliferation, which correlated with lower ERK1/2 phosphorylation. These results suggest a potential tumor suppressor role for C3G-Rap1 pathway in A20 cells.

Rap1 was first identified as a Ras-related gene with suppressor activity against transformation by Ras oncogenes [68], acting by trapping c-Raf in an inactive complex [15, 69]. This inhibitory effect of Rap1 on Ras signaling is not universal, but rather cell-type-dependent [70]. For instance, in T cells, which lack B-Raf, Rap1 interferes with Ras-ERK1/2 signaling at the level of c-Raf, leading to T-cell anergy [13]. However, in myeloid cells, Rap1-GTP directly activates ERKs through B-Raf, independently of Ras [71]. Several lines of evidence also support a tumor suppressor role for C3G in cancer development and progression, playing a negative role in invasion and metastasis in breast cancer [7], CRC [4], HCC [5], and GBM [6], with the latter also showing reduced proliferation. In vitro, C3G suppresses oncogene-induced malignant transformation of NIH-3T3 fibroblasts through a Rap1-independent mechanism, involving the activation of PP2A. This leads to ERK1/2 dephosphorylation, the abrogation of cyclin A expression, and consequently, the inhibition of anchorage-independent growth [16–18].

In contrast, hyperactivation of the C3G-Rap1 pathway has been implicated in the development of hematopoietic malignancies—where oncogenic Ras mutations are rare—primarily based on studies in mouse models with SIPA-1 (also known as SPA-1) deletion, the main Rap1 GAP in hematopoietic cells [13]. In SIPA-1-deficient mice, constitutive Rap1 activation in hematopoietic progenitors leads to a spectrum of myeloid leukemias and, less frequently, T-cell leukemias [72, 73]. Mice transplanted with SIPA-1^⁻/⁻^ hematopoietic stem cells (HSC) additionally expressing membrane-targeted (hyperactive) C3G develop fatal T-cell acute lymphoblastic leukemia due to an abnormal expansion of double-positive thymocytes [74, 75].

This proposed role of C3G-Rap1 axis in leukemogenesis and lymphomagenesis contrasts with our findings in A20 cells. Here we demonstrate that, unlike in myeloid and T cells, C3G acts as a tumor suppressor in the murine B-cell line A20 through both Rap1-dependent and -independent mechanisms. Specifically, C3G hyperactivation led to reduced proliferation, associated with increased Rap1-GTP levels. C3G suppressor activity is probably mediated by Rap1 competitive interference with c-Raf, as described in other cell types [13, 15]. This is supported by the unchanged Ras activation in A20-C3G-Y564H cells, despite their reduced levels of phospho-MEK1/2 and phospho-ERK1/2. In addition, lower levels of all three Raf isoforms were observed in the A20-C3G-Y564H cells, likely further contributing to the suppression of the ERK1/2 pathway. Moreover, no differences in PP2A activity, a mediator of Rap1-independent C3G suppressor activity [18], were observed among the clones. The suppressor activity of C3G on A20 cells was also evident in the increased apoptosis found in the mutant clones, which was linked to lower levels of the anti-apoptotic protein Bcl-xL. Notably, the A20-C3G-Y564H-195 clone exhibited p-ERK1/2 levels comparable to those of the other mutant clones, despite its lower Rap1 activation, suggesting that even a modest increase in Rap1-GTP levels is sufficient to impact the Ras-ERK1/2 pathway.

In contrast to T cells, there is little research examining the role of the C3G-Rap1 axis in B cells. Notably, in vitro assays using A20 cells expressing RapGAPII also demonstrated that Rap1 activation is required for transendothelial migration [29]. However, unlike our findings, that study reported that decreased Rap1/2-GTP levels did not impact cell proliferation. Furthermore, A20 cells overexpressing RapGAPII showed reduced tumor formation in BALB/c mice [29]. One potential explanation for these discrepancies could be differences in C3G levels, which were not assessed in that study. Importantly, unlike models based on gene overexpression or silencing, our system reflects a physiological alteration observed in human lymphomas.

The C3G-Rap1 pathway has also been implicated in the development of the hematopoietic system through sustained activation of the B-Raf-ERK1/2 pathway [71], although no recent data is available. C3G plays a role in the differentiation of multiple cell types, including B cells [1, 25]. Indeed, *Rapgef1* is an essential gene during embryonic development [19]. Since we were unable to obtain C3G-KO clones in Ba/F3 or Boff-p210 cells, we speculate that C3G would also play an essential role during B-cell development.

Adhesion to both the ECM and the endothelium are crucial for leukocyte extravasation and migration [76]. According to the higher Rap1-GTP levels, A20-C3G-Y564H-221 cells exhibited enhanced adhesion to the endothelium, although adhesion to ECM was slightly decreased, likely due to its reduced Rac2 levels. This increase in endothelial adhesion was not observed in the A20-C3G-Y564H-195 clone, which correlated with its lower Rap1 activation (Fig. 2F-H), potentially due to reduced C3G expression in these cells (Figs. 2E and 7B). In parallel with their enhanced adhesion to endothelial cells, A20-C3G-Y564H-221 cells exhibited increased in vitro migration and invasion. This correlated with elevated metastatic potential in BALB/c mice, as evidenced by an increased number of metastatic foci in the liver 10 days after injection, compared to control cells. However, these differences became less pronounced at later time points, likely due to the reduced proliferative capacity of these mutant cells. Surprisingly, despite exhibiting similar in vitro migratory and invasive potential, A20-C3G-Y564H-195 cells demonstrated significantly higher in vivo metastatic activity than the A20-C3G-Y564H-221 clone. This was evidenced by an increased number and size of metastatic foci, resulting in a greater overall tumor burden in the liver. This phenotype may be attributed to the reactivation of ERK1/2 observed in the metastatic liver tissue, potentially mediated by B-Raf; however, the underlying mechanism responsible for the elevated p-ERK1/2 levels remains to be elucidated.

In short, the reduced adhesion observed in the A20-C3G-Y564H-195 clone, likely due to dysregulated Rap1 and Rac2 signaling, may underlie its enhanced motility and invasion. Altered cell adhesion due to changes in C3G expression has also been reported in the chronic myeloid leukemia (CML) cell line K562, likely as a result of aberrant interactions between C3G and FA proteins [49], which are independent of its GEF activity.

The higher expression of *Mmp11* in the A20-C3G-Y564H-195 clone, a matrix metalloproteinase that degrades fibronectin and other substrates [77], may also contribute to its enhanced invasive capacity. Invasion requires cells to breach tissue barriers such as the ECM. Thus, even with impaired adhesion to the ECM, elevated protease activity or a more invasive phenotype could facilitate this process [78]. Notably, matrix metalloproteinases participate in the pro-invasive effect of C3G downregulation in the HCT116 CRC cell line [4].

As mentioned, both mutant clones displayed reduced Rac2 activation (Fig. 5E, F), which may explain their lower adhesion to the ECM. This aligns with the decreased expression of *Dock1* (also known as *Dock180*), a GEF for Rac2 [79, 80], and *Cyria* (*Fam49A*), a regulator of Rac1 activity [81], in both mutant clones compared to the WT clones. DOCK1 is a poly-proline-containing protein that competes with C3G for binding to Crk proteins [79]. Thus, one possible explanation is that hyperactive C3G-Y564H, which is bound to CrkL [82], limits CrkL availability to interact with DOCK1, thereby impairing Rac2 activation. Nonetheless, we cannot exclude the possibility that other Rac2 GEFs, including members of the Dbl family, may also exhibit reduced activity as a result of C3G deregulation.

Rap1 functions as an activator of Rac1 and Rac2 GTPases in various contexts, including in B cells [2, 37]. This supports the notion of a dissociation between C3G and Rap1 in the regulation of A20 cell adhesion to the ECM, as the negative regulation of Rac2 by C3G is unlikely to be mediated by Rap1. It is worth noting that none of these studies evaluated the role of C3G. Interestingly, Rap1-independent regulation of Rac1 by C3G has previously been reported in platelets [36].

The Y554H mutation in the human *RAPGEF1* gene has been associated with the development of non-Hodgkin’s lymphomas [39, 40]. Our results reveal a complex regulatory role for C3G in B-cell lymphoma cells, where both deregulatory mutations and altered C3G expression levels contribute to tumor growth and dissemination.

## Conclusions

In summary, this study demonstrates that C3G exerts a dual role in A20 lymphoma cells through both Rap1-dependent and -independent mechanisms. The C3G-Rap1 axis suppresses proliferation and survival by interfering with the Ras-ERK1/2 pathway, while, independently of Rap1, C3G inhibits Rac2 activity, reducing adhesion to the ECM. In contrast, the C3G-Rap1 pathway enhances adhesion to the endothelium, thereby facilitating migration and homing of A20 metastases. This underscores the complexity of the mechanisms through which C3G regulates various aspects of B-lymphocyte behavior, positioning C3G as a key molecule in B-cell biology.

## Supplementary Information

Additional file 1: This file contains: Tables S1-S3 and Figures S1-S4. 

Additional file 2.

## Data Availability

Data and further details regarding the manuscript are available from the corresponding author on reasonable request.The raw RNA sequencing reads have been deposited in the European Nucleotide Archive (ENA) under accession number PRJEB90154.

## Abbreviations

AIR: Autoinhibitory region
BCR: B-cell receptor
CRC: Colorectal cancer
CXCL12: C-X-C motif chemokine ligand 12
CXCR4: C-X-C chemokine receptor type 4
DEG: Differentially expressed gene
ECL: Enhanced chemiluminescence
ECM: Extracellular matrix
EGM-2: Endothelial growth medium-2
FA: Focal adhesion
FACS: Fluorescence-activated cell sorting
FBS: Fetal bovine serum
GBM: Glioblastoma
gDNA: Genomic DNA
gRNA: Guide RNA
GAP: GTPase activating protein
GEF: Guanine nucleotide exchange factor
HCC: Hepatocellular carcinoma
HDR: Homology directed repair
HRP: Horseradish peroxidase
HUVEC: Human umbilical vein endothelial cell
H&E: Hematoxylin and eosin
IL-3: Interleukin-3
KI: Knock-in
KO: Knock-out
LFA-1: Lymphocyte function-associated antigen 1
MFI: Mean fluorescence intensity
PAM: Protospacer adjacent motif
PI: Propidium iodide
PKC: Protein kinase C
PMA: Phorbol 12-myristate 13-acetate
RFLP: Restriction fragment length polymorphism
SD: Standard deviation
SEM: Standard error of the mean
ssODN: Single-stranded oligodeoxynucleotide
TNF-α: Tumor necrosis factor alpha
VLA-4: Very late antigen-4
WT: Wild-type

## References

[1] Radha V, Mitra A, Dayma K, Sasikumar K. Signalling to actin: role of C3G, a multitasking guanine-nucleotide-exchange factor. Biosci Rep. 2011;31(4):231–44. 21366540 10.1042/BSR20100094

[2] Zhang YL, Wang RC, Cheng K, Ring BZ, Su L. Roles of Rap1 signaling in tumor cell migration and invasion. Cancer Biol Med. 2017;14(1):90–9. 28443208 10.20892/j.issn.2095-3941.2016.0086 PMC5365179

[3] Manzano S, Gutierrez-Uzquiza A, Bragado P, Cuesta AM, Guerrero C, Porras A. C3G protein, a new player in glioblastoma. Int J Mol Sci. 2021. 10.3390/ijms221810018. 34576182 10.3390/ijms221810018 PMC8466177

[4] Priego N, Arechederra M, Sequera C, Bragado P, Vázquez-Carballo A, Gutiérrez-Uzquiza A, et al. C3G knock-down enhances migration and invasion by increasing Rap1-mediated p38α activation, while it impairs tumor growth through p38α-independent mechanisms. Oncotarget. 2016;7:45060–78. 27286263 10.18632/oncotarget.9911 PMC5216706

[5] Sequera C, Bragado P, Manzano S, Arechederra M, Richelme S, Gutierrez-Uzquiza A, et al. C3G is upregulated in hepatocarcinoma, contributing to tumor growth and progression and to HGF/MET pathway activation. Cancers (Basel). 2020;12(8):E2282. 10.3390/cancers12082282 PMC7463771 32823931

[6] Manzano S, Gutierrez-Uzquiza A, Bragado P, Sequera C, Herranz O, Rodrigo-Faus M, et al. C3G downregulation induces the acquisition of a mesenchymal phenotype that enhances aggressiveness of glioblastoma cells. Cell Death Dis. 2021;12(4):348. 33824275 10.1038/s41419-021-03631-w PMC8024353

[7] Dayma K, Radha V. Cytoskeletal remodeling by C3G to induce neurite-like extensions and inhibit motility in highly invasive breast carcinoma cells. Biochim Biophys Acta. 2011;1813(3):456–65. 21223981 10.1016/j.bbamcr.2011.01.004

[8] De Falco V, Castellone MD, De Vita G, Cirafici AM, Hershman JM, Guerrero C, et al. RET/papillary thyroid carcinoma oncogenic signaling through the Rap1 small GTPase. Cancer Res. 2007;67:381–90. 17210721 10.1158/0008-5472.CAN-06-0981

[9] Liu T, Zhang D, Ouyang W, Li R, Wang S, Liu W. Identification and functional characterization of hub genes CLTA, EDIL3, HAPLN1, and HIP1 as diagnostic biomarkers and therapeutic targets in thyroid cancer and Hashimoto’s thyroiditis. Clin Exp Med. 2025;25(1):162. 40372556 10.1007/s10238-025-01689-w PMC12081531

[10] Hirata T, Nagai H, Koizumi K, Okino K, Harada A, Onda M, et al. Amplification, up-regulation and over-expression of C3G (CRK SH3 domain-binding guanine nucleotide-releasing factor) in non-small cell lung cancers. J Hum Genet. 2004;49:290–5. 15138850 10.1007/s10038-004-0148-1

[11] Samuelsson J, Alonso S, Ruiz-Larroya T, Cheung TH, Wong YF, Perucho M. Frequent somatic demethylation of RAPGEF1/C3G intronic sequences in gastrointestinal and gynecological cancer. Int J Oncol. 2011;38:1575–7. 21399874 10.3892/ijo.2011.972

[12] Okino K, Nagai H, Nakayama H, Doi D, Yoneyama K, Konishi H, et al. Inactivation of Crk SH3 domain-binding guanine nucleotide-releasing factor (C3G) in cervical squamous cell carcinoma. Int J Gynecol Cancer. 2006;16(2):763–71. 16681758 10.1111/j.1525-1438.2006.00352.x

[13] Kometani K, Ishida D, Hattori M, Minato N. Rap1 and SPA-1 in hematologic malignancy. Trends Mol Med. 2004;10:401–8. 15310461 10.1016/j.molmed.2004.06.004

[14] Gutierrez-Berzal J, Castellano E, Martin-Encabo S, Gutierrez-Cianca N, Hernandez JM, Santos E, et al. Characterization of p87C3G, a novel, truncated C3G isoform that is overexpressed in chronic myeloid leukemia and interacts with Bcr-Abl. Exp Cell Res. 2006;312(6):938–48. 16443220 10.1016/j.yexcr.2005.12.007

[15] Sakoda T, Kaibuchi K, Kishi K, Kishida S, Doi K, Hoshino M, et al. smg/rap1/Krev-1 p21s inhibit the signal pathway to the c-fos promoter/enhancer from c-Ki-ras p21 but not from c-raf-1 kinase in NIH3T3 cells. Oncogene. 1992;7:1705–11. 1323817

[16] Guerrero C, Fernandez-Medarde A, Rojas JM, Font de Mora J, Esteban LM, Santos E. Transformation suppressor activity of C3G is independent of its CDC25-homology domain. Oncogene. 1998;16(5):613–24. 9482107 10.1038/sj.onc.1201569

[17] Guerrero C, Martin-Encabo S, Fernandez-Medarde A, Santos E. C3G-mediated suppression of oncogene-induced focus formation in fibroblasts involves inhibition of ERK activation, cyclin A expression and alterations of anchorage-independent growth. Oncogene. 2004;23(28):4885–93. 15077165 10.1038/sj.onc.1207622

[18] Martín-Encabo S, Santos E, Guerrero C. C3G mediated suppression of malignant transformation involves activation of PP2A phosphatases at the subcortical actin cytoskeleton. Exp Cell Res. 2007;313(18):3881–91. 17825818 10.1016/j.yexcr.2007.07.036

[19] Ohba Y, Ikuta K, Ogura A, Matsuda J, Mochizuki N, Nagashima K, et al. Requirement for C3G-dependent Rap1 activation for cell adhesion and embryogenesis. EMBO J. 2001;20(13):3333–41. 11432821 10.1093/emboj/20.13.3333 PMC125518

[20] Voss AK, Gruss P, Thomas T. The guanine nucleotide exchange factor C3G is necessary for the formation of focal adhesions and vascular maturation. Development. 2003;130:355–67. 12466202 10.1242/dev.00217

[21] Palao N, Sequera C, Cuesta AM, Baquero C, Bragado P, Gutierrez-Uzquiza A, et al. C3G down-regulation enhances pro-migratory and stemness properties of oval cells by promoting an epithelial-mesenchymal-like process. Int J Biol Sci. 2022;18(15):5873–84. 36263169 10.7150/ijbs.73192 PMC9576514

[22] Ortiz-Rivero S, Baquero C, Hernandez-Cano L, Roldan-Etcheverry JJ, Gutierrez-Herrero S, Fernandez-Infante C, et al. C3G, through its GEF activity, induces megakaryocytic differentiation and proplatelet formation. Cell Commun Signal. 2018;16(1):101. 30567575 10.1186/s12964-018-0311-5 PMC6299959

[23] Hernández-Cano L, Fernández-Infante C, Herranz O, Berrocal P, Lozano FS, Sánchez-Martín MA, et al. New functions of C3G in platelet biology: contribution to ischemia-induced angiogenesis, tumor metastasis and TPO clearance. Front Cell Dev Biol. 2022;10:1026287. 36393850 10.3389/fcell.2022.1026287 PMC9661425

[24] Vishnu VV, Muralikrishna B, Verma A, Nayak SC, Sowpati DT, Radha V, et al. C3G regulates STAT3, ERK, adhesion signaling, and is essential for differentiation of embryonic stem cells. Stem Cell Rev Rep. 2021;17(4):1465–77. 33624208 10.1007/s12015-021-10136-8 PMC8372029

[25] Smit L, van der Horst G, Borst J. Sos, Vav, and C3G participate in B cell receptor-induced signaling pathways and differentially associate with Shc-Grb2, Crk, and Crk-L adaptors. J Biol Chem. 1996;271:8564–9. 8621483 10.1074/jbc.271.15.8564

[26] Chu H, Awasthi A, White GC, Chrzanowska-Wodnicka M, Malarkannan S. Rap1b regulates B cell development, homing, and T cell-dependent humoral immunity. J Immunol. 2008;181(5):3373–83. 18714009 10.4049/jimmunol.181.5.3373 PMC4571460

[27] Lin KB, Freeman SA, Zabetian S, Brugger H, Weber M, Lei V, et al. The rap GTPases regulate B cell morphology, immune-synapse formation, and signaling by particulate B cell receptor ligands. Immunity. 2008;28(1):75–87. 18191594 10.1016/j.immuni.2007.11.019

[28] McLeod SJ, Li AH, Lee RL, Burgess AE, Gold MR. The Rap GTPases regulate B cell migration toward the chemokine stromal cell-derived factor-1 (CXCL12): potential role for Rap2 in promoting B cell migration. J Immunol. 2002;169(3):1365–71. 12133960 10.4049/jimmunol.169.3.1365

[29] Lin KB, Tan P, Freeman SA, Lam M, McNagny KM, Gold MR. The Rap GTPases regulate the migration, invasiveness and in vivo dissemination of B-cell lymphomas. Oncogene. 2010;29(4):608–15. 19838206 10.1038/onc.2009.345

[30] Cook DR, Rossman KL, Der CJ. Rho guanine nucleotide exchange factors: regulators of Rho GTPase activity in development and disease. Oncogene. 2014;33:4021–35. 24037532 10.1038/onc.2013.362 PMC4875565

[31] Shirsat NV, Pignolo RJ, Kreider BL, Rovera G. A member of the Ras gene superfamily is expressed specifically in T, B and myeloid hemopoietic cells. Oncogene. 1990;5(5):769–72. 2189110

[32] Kazanietz MG, Caloca MJ. The Rac GTPase in cancer: from old concepts to new paradigms. Cancer Res. 2017;77(20):5445–51. 28807941 10.1158/0008-5472.CAN-17-1456 PMC5645227

[33] Takahashi M, Rikitake Y, Nagamatsu Y, Hara T, Ikeda W, Hirata K, et al. Sequential activation of Rap1 and Rac1 small G proteins by PDGF locally at leading edges of NIH3T3 cells. Genes Cells. 2008;13(6):549–69. 18422604 10.1111/j.1365-2443.2008.01187.x

[34] Williams JA, Chen X, Sabbatini ME. Small G proteins as key regulators of pancreatic digestive enzyme secretion. Am J Physiol Endocrinol Metab. 2009;296(3):E405–14. 19088252 10.1152/ajpendo.90874.2008 PMC2660147

[35] Stefanini L, Boulaftali Y, Ouellette TD, Holinstat M, Désiré L, Leblond B, et al. Rap1-Rac1 circuits potentiate platelet activation. Arterioscler Thromb Vasc Biol. 2012;32:434–41. 22075250 10.1161/ATVBAHA.111.239194 PMC3262085

[36] Fernández-Infante C, Hernández-Cano L, Herranz O, Berrocal P, Sicilia-Navarro C, González-Porras JR, et al. Platelet C3G: a key player in vesicle exocytosis, spreading and clot retraction. Cell Mol Life Sci. 2024;81(1):84. 38345631 10.1007/s00018-023-05109-8 PMC10861696

[37] Arana E, Vehlow A, Harwood NE, Vigorito E, Henderson R, Turner M, et al. Activation of the small GTPase Rac2 via the B cell receptor regulates B cell adhesion and immunological-synapse formation. Immunity. 2008;28(1):88–99. 18191593 10.1016/j.immuni.2007.12.003

[38] Carabias A, Gomez-Hernandez M, de Cima S, Rodriguez-Blazquez A, Moran-Vaquero A, Gonzalez-Saenz P, et al. Mechanisms of autoregulation of C3G, activator of the GTPase Rap1, and its catalytic deregulation in lymphomas. Sci Signal. 2020;13(647):eabb7075. 32873726 10.1126/scisignal.abb7075

[39] Morin RD, Mendez-Lago M, Mungall AJ, Goya R, Mungall KL, Corbett RD, et al. Frequent mutation of histone-modifying genes in non-Hodgkin lymphoma. Nature. 2011;476(7360):298–303. 21796119 10.1038/nature10351 PMC3210554

[40] Green MR, Gentles AJ, Nair RV, Irish JM, Kihira S, Liu CL, et al. Hierarchy in somatic mutations arising during genomic evolution and progression of follicular lymphoma. Blood. 2013;121(9):1604–11. 23297126 10.1182/blood-2012-09-457283 PMC3587323

[41] Schindelin J, Arganda-Carreras I, Frise E, Kaynig V, Longair M, Pietzsch T, et al. Fiji: an open-source platform for biological-image analysis. Nat Methods. 2012;9(7):676–82. 22743772 10.1038/nmeth.2019 PMC3855844

[42] Stockert JC, Horobin RW, Colombo LL, Blazquez-Castro A. Tetrazolium salts and formazan products in cell biology: viability assessment, fluorescence imaging, and labeling perspectives. Acta Histochem. 2018;120(3):159–67. 29496266 10.1016/j.acthis.2018.02.005

[43] Livak KJ, Schmittgen TD. Analysis of relative gene expression data using real-time quantitative PCR and the 2(-Delta delta C(T)) method. Methods. 2001;25(4):402–8. 11846609 10.1006/meth.2001.1262

[44] Liao Y, Smyth GK, Shi W. Featurecounts: an efficient general purpose program for assigning sequence reads to genomic features. Bioinf (Oxford England). 2014;30(7):923–30. 10.1093/bioinformatics/btt656 24227677

[45] Love MI, Huber W, Anders S. Moderated estimation of fold change and dispersion for RNA-seq data with DESeq2. Genome Biol. 2014;15(12):550. 25516281 10.1186/s13059-014-0550-8 PMC4302049

[46] Yu G, Wang LG, Han Y, He QY. Clusterprofiler: an R package for comparing biological themes among gene clusters. OMICS. 2012;16(5):284–7. 22455463 10.1089/omi.2011.0118 PMC3339379

[47] Carabias A, Guerrero C, de Pereda JM. C3G self-regulatory mechanism revealed: implications for hematopoietic malignancies. Mol Cell Oncol. 2021;8(1):e1837581. 10.1080/23723556.2020.1837581 PMC7849780 33553598

[48] Maia V, Sanz M, Gutierrez-Berzal J, de Luis A, Gutierrez-Uzquiza A, Porras A, et al. C3G Silencing enhances STI-571-induced apoptosis in CML cells through p38 MAPK activation, but it antagonizes STI-571 inhibitory effect on survival. Cell Signal. 2009;21(7):1229–35. 19324082 10.1016/j.cellsig.2009.03.015

[49] Maia V, Ortiz-Rivero S, Sanz M, Gutierrez-Berzal J, Alvarez-Fernández I, Gutierrez-Herrero S, et al. C3G forms complexes with Bcr-Abl and p38alpha MAPK at the focal adhesions in chronic myeloid leukemia cells: implication in the regulation of leukemic cell adhesion. Cell Commun Signal. 2013;11(1):9. 23343344 10.1186/1478-811X-11-9 PMC3629710

[50] Hanahan D, Weinberg RA. The hallmarks of cancer. Cell. 2000;100:57–70. 10647931 10.1016/s0092-8674(00)81683-9

[51] Shivakrupa R, Radha V, Sudhakar C, Swarup G. Physical and functional interaction between Hck tyrosine kinase and guanine nucleotide exchange factor C3G results in apoptosis, which is independent of C3G catalytic domain. J Biol Chem. 2003;278:52188–94. 14551197 10.1074/jbc.M310656200

[52] Yang D, Zhang L, Zhang Z, Hu S, Fu Y, Laukkanen JA, et al. Silencing of C3G increases cardiomyocyte survival inhibition and apoptosis via regulation of p-ERK1/2 and Bax. Clin Exp Pharmacol Physiol. 2019;46(3):237–45. 30152875 10.1111/1440-1681.13027

[53] Gutiérrez-Uzquiza A, Arechederra M, Molina I, Baños R, Maia V, Benito M, et al. C3G down-regulates p38 MAPK activity in response to stress by Rap-1 independent mechanisms: involvement in cell death. Cell Signal. 2010;22(3):533–42. 19925863 10.1016/j.cellsig.2009.11.008

[54] Lavoie H, Gagnon J, Therrien M. ERK signalling: a master regulator of cell behaviour, life and fate. Nat Rev Mol Cell Biol. 2020;21(10):607–32. 32576977 10.1038/s41580-020-0255-7

[55] Wellbrock C, Karasarides M, Marais R. The RAF proteins take centre stage. Nat Rev Mol Cell Biol. 2004;5(11):875–85. 15520807 10.1038/nrm1498

[56] Brant R, Sharpe A, Liptrot T, Dry JR, Harrington EA, Barrett JC, et al. Clinically viable gene expression assays with potential for predicting benefit from MEK inhibitors. Clin Cancer Res. 2017;23(6):1471–80. 27733477 10.1158/1078-0432.CCR-16-0021

[57] Humphries JD, Byron A, Humphries MJ. Integrin ligands at a glance. J Cell Sci. 2006;119(Pt 19):3901–3. 16988024 10.1242/jcs.03098 PMC3380273

[58] Nagy-Balo Z, Kiss R, Menge A, Bodor C, Bajtay Z, Erdei A. Activated human memory B lymphocytes use CR4 (CD11c/CD18) for adhesion, migration, and proliferation. Front Immunol. 2020;11:565458. 33133077 10.3389/fimmu.2020.565458 PMC7550640

[59] Sun H, Lagarrigue F, Ginsberg MH. The connection between Rap1 and Talin1 in the activation of integrins in blood cells. Front Cell Dev Biol. 2022;10:908622. 35721481 10.3389/fcell.2022.908622 PMC9198492

[60] Tybulewicz VL, Henderson RB. Rho family GTPases and their regulators in lymphocytes. Nat Rev Immunol. 2009;9(9):630–44. 19696767 10.1038/nri2606 PMC4898593

[61] Lawson CD, Ridley AJ. Rho GTPase signaling complexes in cell migration and invasion. J Cell Biol. 2018;217(2):447–57. 29233866 10.1083/jcb.201612069 PMC5800797

[62] Passineau MJ, Siegal GP, Everts M, Pereboev A, Jhala D, Wang M, et al. The natural history of a novel, systemic, disseminated model of syngeneic mouse B-cell lymphoma. Leuk Lymphoma. 2005;46(11):1627–38. 16334907 10.1080/10428190500221454x

[63] Gutiérrez-Herrero S, Maia V, Gutiérrez-Berzal J, Calzada N, Sanz M, González-Manchón C, et al. C3G transgenic mouse models with specific expression in platelets reveal a new role for C3G in platelet clotting through its GEF activity. Biochimica et Biophysica Acta (BBA). 2012;1823(8):1366–77. 22659131 10.1016/j.bbamcr.2012.05.021

[64] Martín-Granado V, Ortiz-Rivero S, Carmona R, Gutiérrez-Herrero S, Barrera M, San-Segundo L, et al. C3G promotes a selective release of angiogenic factors from activated mouse platelets to regulate angiogenesis and tumor metastasis. Oncotarget. 2017;8(67):110994–1011. 29340032 10.18632/oncotarget.22339 PMC5762300

[65] Gutiérrez-Herrero S, Fernandez-Infante C, Hernandez-Cano L, Ortiz-Rivero S, Guijas C, Martin-Granado V, et al. C3G contributes to platelet activation and aggregation by regulating major signaling pathways. Signal Transduct Target Ther. 2020;5:29. 32296045 10.1038/s41392-020-0119-9 PMC7109025

[66] Herranz O, Berrocal P, Sicilia-Navarro C, Fernandez-Infante C, Hernandez-Cano L, Porras A, et al. C3G promotes bone marrow adipocyte expansion and hematopoietic regeneration after myeloablation by enhancing megakaryocyte niche function. J Hematol Oncol. 2025;18(1):38. 40170099 10.1186/s13045-025-01687-1 PMC11959767

[67] Kawamata N, Pennella MA, Woo JL, Berk AJ, Koeffler HP. Dominant-negative mechanism of leukemogenic PAX5 fusions. Oncogene. 2012;31(8):966–77. 21765475 10.1038/onc.2011.291 PMC3197879

[68] Kitayama H, Sugimoto Y, Matsuzaki T, Ikawa Y, Noda M. A ras-related gene with transformation suppressor activity. Cell. 1989;56:77–84. 2642744 10.1016/0092-8674(89)90985-9

[69] Hu CD, Kariya K, Kotani G, Shirouzu M, Yokoyama S, Kataoka T. Coassociation of Rap1A and Ha-Ras with Raf-1 N-terminal region interferes with ras-dependent activation of Raf-1. J Biol Chem. 1997;272(18):11702–5. 9115221 10.1074/jbc.272.18.11702

[70] Bos JL, de Rooij J, Reedquist KA. Rap1 signalling: adhering to new models. Nat Rev Mol Cell Biol. 2001;2:369–77. 11331911 10.1038/35073073

[71] Stork PJ, Dillon TJ. Multiple roles of Rap1 in hematopoietic cells: complementary versus antagonistic functions. Blood. 2005;106:2952–61. 16076873 10.1182/blood-2005-03-1062 PMC1895320

[72] Minato N, Hattori M. Spa-1 (Sipa1) and Rap signaling in leukemia and cancer metastasis. Cancer Sci. 2009;100:17–23. 19037996 10.1111/j.1349-7006.2008.01011.x PMC11158263

[73] Xiao P, Dolinska M, Sandhow L, Kondo M, Johansson AS, Bouderlique T, et al. Sipa1 deficiency-induced bone marrow niche alterations lead to the initiation of myeloproliferative neoplasm. Blood Adv. 2018;2(5):534–48. 29514790 10.1182/bloodadvances.2017013599 PMC5851419

[74] Wang SF, Aoki M, Nakashima Y, Shinozuka Y, Tanaka H, Taniwaki M, et al. Development of notch-dependent T-cell leukemia by deregulated Rap1 signaling. Blood. 2008;111:2878–86. 18180377 10.1182/blood-2007-07-103119

[75] Minato N. Rap G protein signal in normal and disordered lymphohematopoiesis. Exp Cell Res. 2013;319(15):2323–8. 23603280 10.1016/j.yexcr.2013.04.009

[76] Hordijk PL. Recent insights into endothelial control of leukocyte extravasation. Cell Mol Life Sci. 2016;73(8):1591–608. 26794844 10.1007/s00018-016-2136-y PMC11108429

[77] Zhang X, Huang S, Guo J, Zhou L, You L, Zhang T, et al. Insights into the distinct roles of MMP-11 in tumor biology and future therapeutics (Review). Int J Oncol. 2016;48(5):1783–93. 26892540 10.3892/ijo.2016.3400

[78] Cui N, Hu M, Khalil RA. Biochemical and biological attributes of matrix metalloproteinases. Prog Mol Biol Transl Sci. 2017;147:1–73. 28413025 10.1016/bs.pmbts.2017.02.005 PMC5430303

[79] Valles AM, Beuvin M, Boyer B. Activation of Rac1 by paxillin-Crk-DOCK180 signaling complex is antagonized by Rap1 in migrating NBT-II cells. J Biol Chem. 2004;279:44490–6. 15308668 10.1074/jbc.M405144200

[80] Meyer AE, Stelloh C, Pulakanti K, Burns R, Fisher JB, Heimbruch KE, et al. Combinatorial genetics reveals the Dock1-Rac2 axis as a potential target for the treatment of NPM1;Cohesin mutated AML. Leukemia. 2022;36(8):2032–41. 35778533 10.1038/s41375-022-01632-y PMC9357218

[81] Le AH, Yelland T, Paul NR, Fort L, Nikolaou S, Ismail S, et al. CYRI-A limits invasive migration through macropinosome formation and integrin uptake regulation. J Cell Biol. 2021. 10.1083/jcb.202012114. 34165494 10.1083/jcb.202012114 PMC8236918

[82] Rodriguez-Blazquez A, Carabias A, Moran-Vaquero A, de Cima S, Luque-Ortega JR, Alfonso C, et al. Crk proteins activate the Rap1 guanine nucleotide exchange factor C3G by segregated adaptor-dependent and -independent mechanisms. Cell Commun Signal. 2023;21(1):30. 36737758 10.1186/s12964-023-01042-2 PMC9896810

