# Supplementary material for: C3G deregulation uncovers a dual role in B-cell lymphoma: tumor suppression and enhanced metastasis via Rap1 and Rac2 signaling
Source: Cell Commun Signal. 2025 Nov 27;24:11. doi: 10.1186/s12964-025-02551-y (PMC12781495; doi:10.1186/s12964-025-02551-y)
Supplement: Supplementary file 1 — Additional file 1: This file contains: Tables S1-S3 and Figures S1-S4. [file 12964_2025_2551_MOESM1_ESM.pdf]

Additional file 1

C3G deregulation uncovers a dual role in B-cell lymphoma: tumor suppression and enhanced metastasis via Rap1 and Rac2 signaling

Running title: C3G dual function in B-cell lymphoma

Alba Morán-Vaquero[1,2,3], Óscar Herranz[1,2,3], Ana Dávila-Hidalgo[1,2,3], Antonio Rodríguez-Blázquez[1,2,3], Cristina Fernández-Infante[1,2,3], Ignacio García-Tuñón[1,2,4], Elena Vuelta[1,2,4], Femke van der Meer[5], Coert Margadant[5], Carmen Guerrero[1,2,3]* and José M. de Pereda[1]*

[1]Centro de Investigación del Cáncer, Consejo Superior de Investigaciones Científicas (CSIC), Universidad de Salamanca, 37007 Salamanca, Spain.
[2]Instituto de Investigación Biomédica de Salamanca (IBSAL), Salamanca, Spain.
[3]Departamento de Medicina, Universidad de Salamanca, Salamanca, Spain.
[4]Departamento de Biomedicina y Biotecnología. Universidad de Alcalá, Alcalá de Henares, Spain.
[5]Institute of Biology, Leiden University, Gorleaus Building, Einsteinweg 55, 2333 CC Leiden, The Netherlands.

*Correspondence and equal contribution: C. Guerrero and JM. de Pereda, Centro de Investigación del Cáncer, Campus Unamuno s/n, Salamanca, Spain. Tel.: +34 923294801; e-mail: cguerrero@usal.es; jm.depereda@csic.es

Supplementary Tables

Table S1. Primary antibodies used in western blot.

Antibody	Host	Supplier	Reference	RRID	Dilution
β-actin	Mouse	Sigma-Aldrich	A5441	AB_476744	1:1000
β-tubulin (2-28-33)	Mouse	Sigma-Aldrich	T5293	AB_477580	1:1000
Bcl-xL	Rabbit	Cell Signaling	2762	AB_10694844	1:1000
C3G (F-5)	Mouse	Santa Cruz Biotechnology	sc-376992	n.a.	1:1000
C3G (G-9)	Mouse	Santa Cruz Biotechnology	sc-393836	n.a.	1:1000
C3G (G-4)	Mouse	Santa Cruz Biotechnology	sc-17840	AB_628203	1:1000
Cdc42	Rabbit	Proteintech	10155-1-AP	AB_2078096	1:1000
ERK1 (K-23)	Rabbit	Santa Cruz Biotechnology	sc-94	AB_2140110	1:1000
p-ERK (E-4)	Mouse	Santa Cruz Biotechnology	sc-7383	AB_627545	1:1000
GFP (FL)	Rabbit	Santa Cruz Biotechnology	sc-8334	AB_641123	1:1000
GST (B-14)	Mouse	Santa Cruz Biotechnology	sc-138	AB_627677	1:1000
polyHis	Mouse	Sigma-Aldrich	H1029	AB_260015	1:1000
MEK1/2	Rabbit	Cell Signaling	9122	AB_823567	1:1000
phospho-MEK1/2	Rabbit	Cell Signaling	9121	AB_331648	1:1000
PP2A, C sub (1D6)	Mouse	Merck	05-421	AB_309726	1:1000
Rac1	Mouse	Cytoskeleton	ARC03	AB_10709099	1:500
Rac2	Rabbit	Proteintech	10735-1-AP	AB_2176127	1:1000
c-Raf (D43BJ)	Rabbit	Cell Signaling	53745	AB_2799444	1:1000
Rap1 (E-6)	Mouse	Santa Cruz Biotechnology	sc-398755	AB_2884025	1:800
pan-Ras (C-4)	Mouse	Santa Cruz Biotechnology	sc-166691	AB_2154229	1:1000
Talin (C-20)	Goat	Santa Cruz Biotechnology	sc-7534	AB_661610	1:500

n.a.: Not available

Table S2. Secondary antibodies used in western blot.

Antibody	Host	Supplier	Reference	RRID	Dilution	Detection
anti-mouse Ig (H+L) DyLight 800	Goat	ThermoFisher	SA5-35521	AB_2556774	1:5000	Odyssey
anti-mouse IgG (H+L) DyLight 680	Goat	ThermoFisher	SA5-35518	AB_614942	1:5000	Odyssey
anti-rabbit IgG (H+L) DyLight 800	Goat	ThermoFisher	SA5-10036	AB_2556616	1:10000	Odyssey
anti-rabbit IgG (H+L) DyLight 680	Goat	ThermoFisher	SA5-35568	AB_614946	1:10000	Odyssey
anti-goat AF680 IgG (H+L)	Donkey	ThermoFisher	A-21084	AB_2535741	1:5000	Odyssey
anti-mouse IgG-HRP	Sheep	Cytiva	NXA931	AB_772209	1:5000	ECL
anti-rabbit IgG-HRP	Goat	Santa Cruz Biotechnology	sc-2004	AB_631746	1:10000	ECL

Table S3. Sequence of forward and reverse primers used in RT-qPCR to quantify the expression of the indicated mouse genes.

Gene	Forward primer (5'-3')	Reverse primer (5'-3')
N-terminal *Rapgef1*	GTGAGCAAAGAGGCAAGAGA	CACAGCACTGGTGGACATAA
C-terminal *Rapgef1*	ATTTCCACAGCCACGAGATAG	CTCTTCTCCTCATTCTGCTCTT
Araf	TGGCGATGTAGCTGTGAAAG	CTGTGTGATGATGGCAAACC
Braf	ATGCCCTTCAGCAAAGAGAA	ATCTTGCGGGTACCACTGTC
Craf	CTACACCCCATGCCTTCACT	GCTGAAGGTGAGGCTGATTC
Phlda1	GGGCTACTGCTCATACCGC	AAAAGTGCAATTCCTTCAGCTTG
Spry2	TCCAAGAGATGCCCTTACCCA	GCAGACCGTGGAGTCTTTCA
Dusp6	GCGTCGGAAATGGCGATCT	ATGTGTGACGACTCGTACAGC
Dusp4	CGTGCGCTGCAATACCATC	CTCATAGCCACCTTTAAGCAGG
Etv4	CGGAGGATGAAAGGCGGATAC	TCTTGGAAGTGACTGAGGTCC
Etv5	TCAGTCTGATAACTTGGTGCTTC	GGCTTCCTATCGTAGGCACAA
Dock1	CAGGAAGCATAAATACCTCGCC	CAGCTCATCCGATTGTCTTTGT
Cyria	GAGCGAGAGATATGGAACCAGA	AGTGCTTTTTCAAGACGGATGG
Armcx3	CTGGAGCCTGCTATTGCATTT	TCAGACCAGTCATTATACCTGGC
Armcx6	TGGGAAGAAGTGAGGGGAAC	GTCGAGCCATTGCTGTGAAAT
Nol4l	GGACCAGCCCATTAACCTGT	GCTGTATGGGGGTGACTCG
Actb	CCACCATGTACCCAGGCATT	CAGCTCAGTAACAGTCCGCC

Supplementary figures

Figure S1

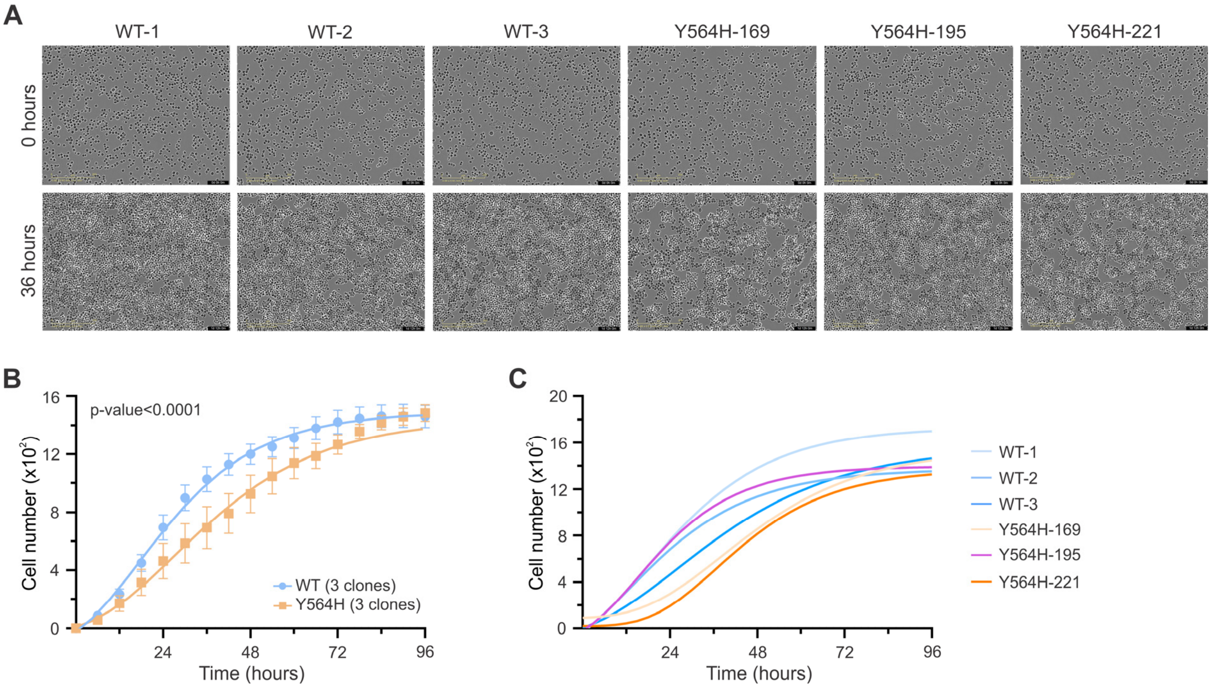

Figure S1. C3G-Y564H mutation slows down A20 cell proliferation. The number of live cells was quantified using live-cell imaging of label-free cell module with an Incucyte SX5 instrument (Sartorius). Images were captured at 6-hour intervals over 4 days, analyzing three A20-C3G-WT and three A20-C3G-Y564H cell clones. (**A**) Representative images at 0 hours (upper panels) confirm the initial seeding density, while images at 36 hours (lower panels) highlight the most pronounced differences among clones. (**B**) Time course of cell numbers presented as the mean ± SEM of grouped data from three A20-C3G-WT and three A20-C3G-Y564H clones. Images were processed using the Non-Adherent Cell-by-Cell Analysis software module. Lines represent a 4-parameter Gompertz growth model fitted to the experimental data. (**C**) Growth curves of individual clones, each fitted with a 4-parameter Gompertz growth model. Two independent experiments were performed per clone, with experimental triplicates and four images taken per condition.

Figure S2

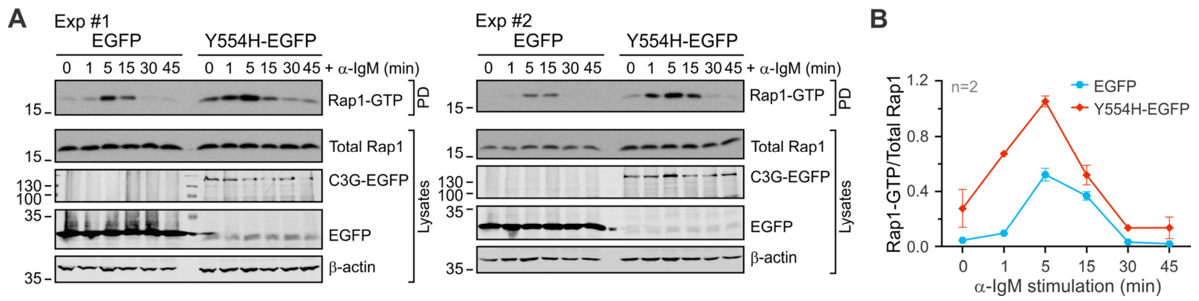

Figure S2. **Expression of C3G-Y554H in A20 cells increases Rap1 activation**. A20 cells were electroporated with the pEF1-C3GY554H-EGFP construct to generate C3G-Y554H-expressing clones. (**A**) Two Rap1-GTP pull-down experiments are shown, including C3G protein levels. (**B**) Quantification of Rap1-GTP levels demonstrates an increase in C3G-Y554H-overexpressing A20 cells compared with control cells.

Figure S3

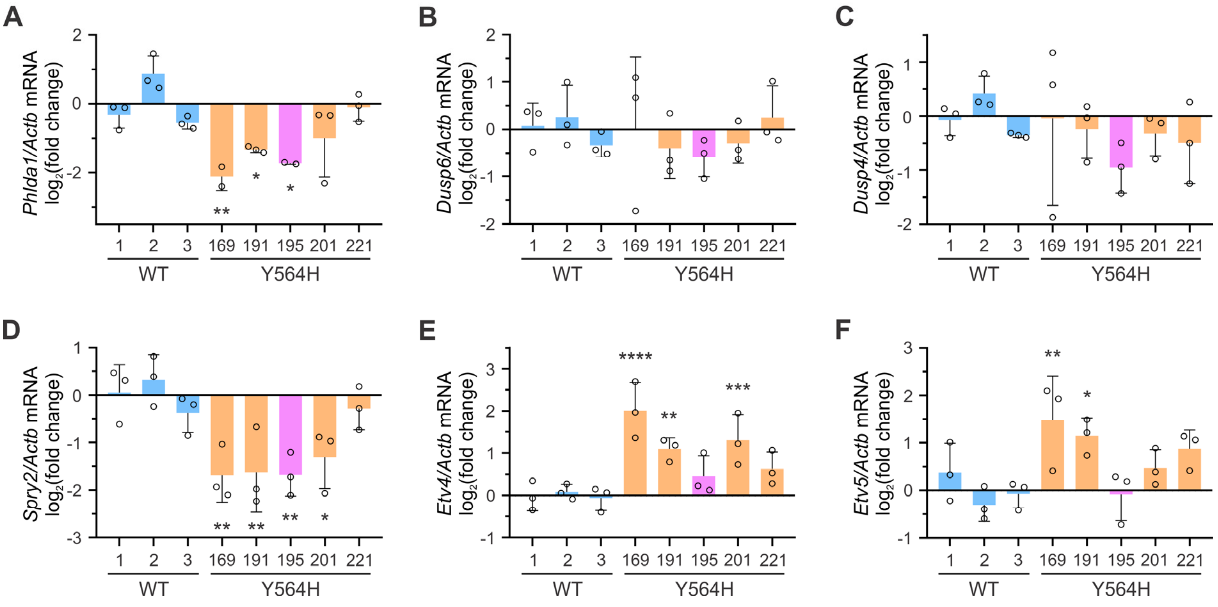

Figure S3. A20-C3G-Y564H cells display altered expression of ERK1/2-regulated genes. mRNA expression levels of (**A**) *Phlda1*, (**B**) *Dusp6*, (**C**) *Dusp4*, (**D**) *Spry2*, (**E**) *Etv4*, and (**F**) *Etv5* were analyzed in A20-C3G-Y564H and A20-C3G-WT clones by RT-qPCR. Gene expression levels were relative to *Actb*. Bar charts represent the mean ± SEM of the $\log_2$(fold change) from three independent experiments, with individual data points shown. *p<0.05, **p<0.01, ***p<0.001, ****p<0.0001.

Figure S4

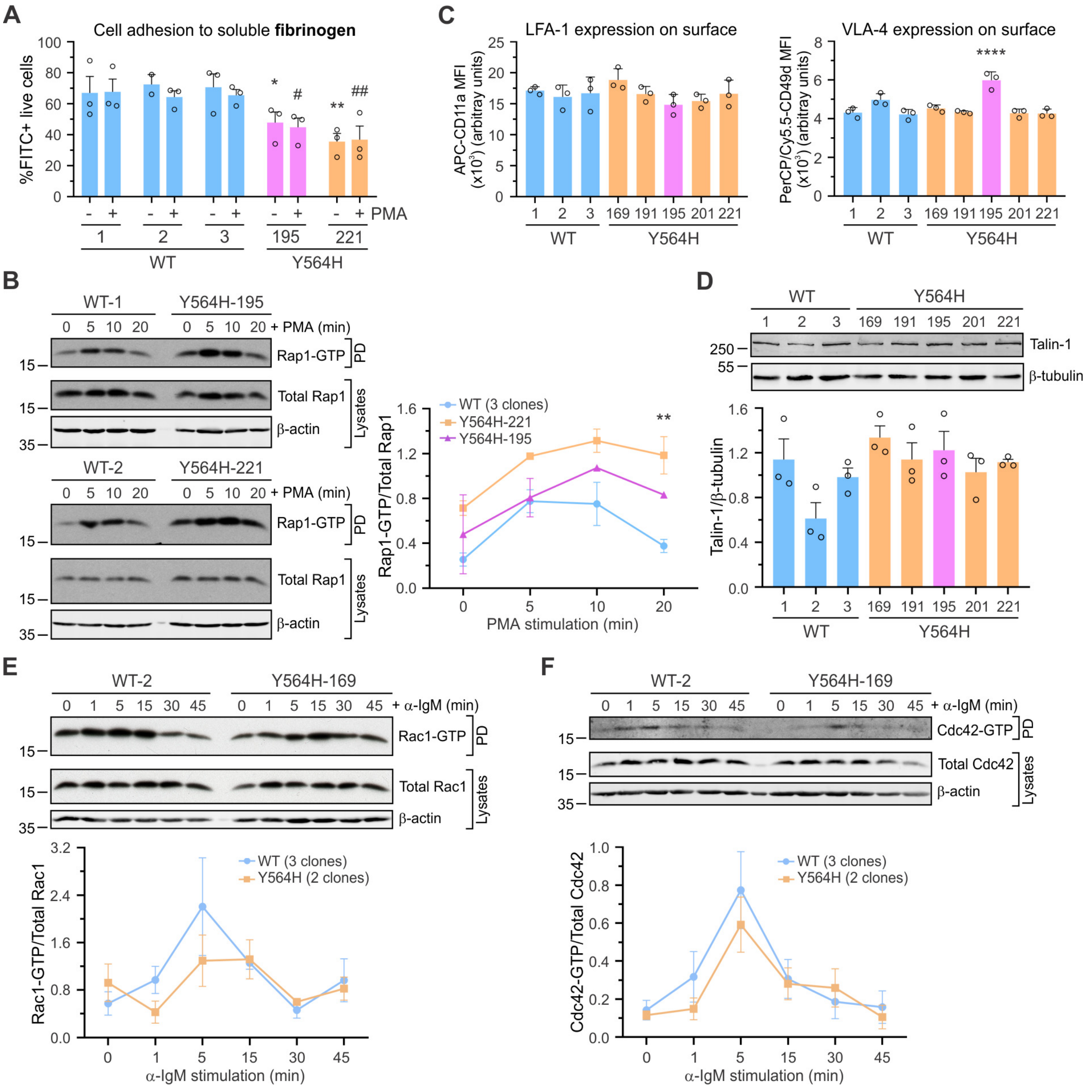

Figure S4. A20-C3G-Y564H cells exhibit reduced fibrinogen binding with no changes in LFA-1, VLA-4, or Talin-1 levels or in Rac1 and Cdc42 activity. (**A**) Cells were stimulated with 20 nM PMA and incubated with Alexa Fluor 488-conjugated human fibrinogen. The bar chart represents the mean ± SEM of the percentage of live cells bound to soluble fibrinogen (FITC+). Three independent experiments were performed with each clone. **p*<0.05, ***p*<0.01 versus unstimulated A20-C3G-WT cells; #*p*<0.05, ##*p*<0.01 versus PMA-stimulated A20-C3G-WT cells. (**B**) Rap1 activation time course after stimulation with 10 ng/mL PMA. Rap1-GTP level were detected by pull-down followed by western blot. Total Rap1 and β-actin expression were analyzed as references. Graphs represents the mean ± SEM of grouped data from three A20-C3G-WT and the indicated A20-C3G-Y564H clones, with at least two experiments in each case. ***p*<0.01 when comparing A20-C3G-WT with A20-C3G-Y564H-221 cells.

(**C**) Flow cytometry analysis of LFA-1 (αLβ2) (left) and VLA-4 (α4β1) (right) integrin levels on the cell surface using anti-mouse APC-CD11a and anti-mouse PerCP/Cyanine5.5-CD49d conjugated antibodies, respectively. Bar charts represent the mean ± SD of the mean fluorescence intensity (MFI) from three independent experiments. ****p<0.0001. (**D**) Talin-1 protein levels were analyzed by western blot. A representative experiment is shown. The bar chart represents the mean ± SEM of Talin-1 levels, relativized to β-tubulin, from three independent experiments. (**E, F**) A20 cells were stimulated with 10 μg/ml anti-IgM, and (**E**) Rac1-GTP or (**F**) Cdc42-GTP levels were detected at different times. Representative experiments are shown. Total Rac1, Cdc42, and β-actin levels in cell lysates were used as loading controls. Graphs represents the mean ± SEM of grouped data from three A20-C3G-WT and two A20-C3G-Y564H clones, with at least one experiment performed per clone.

Figure S5

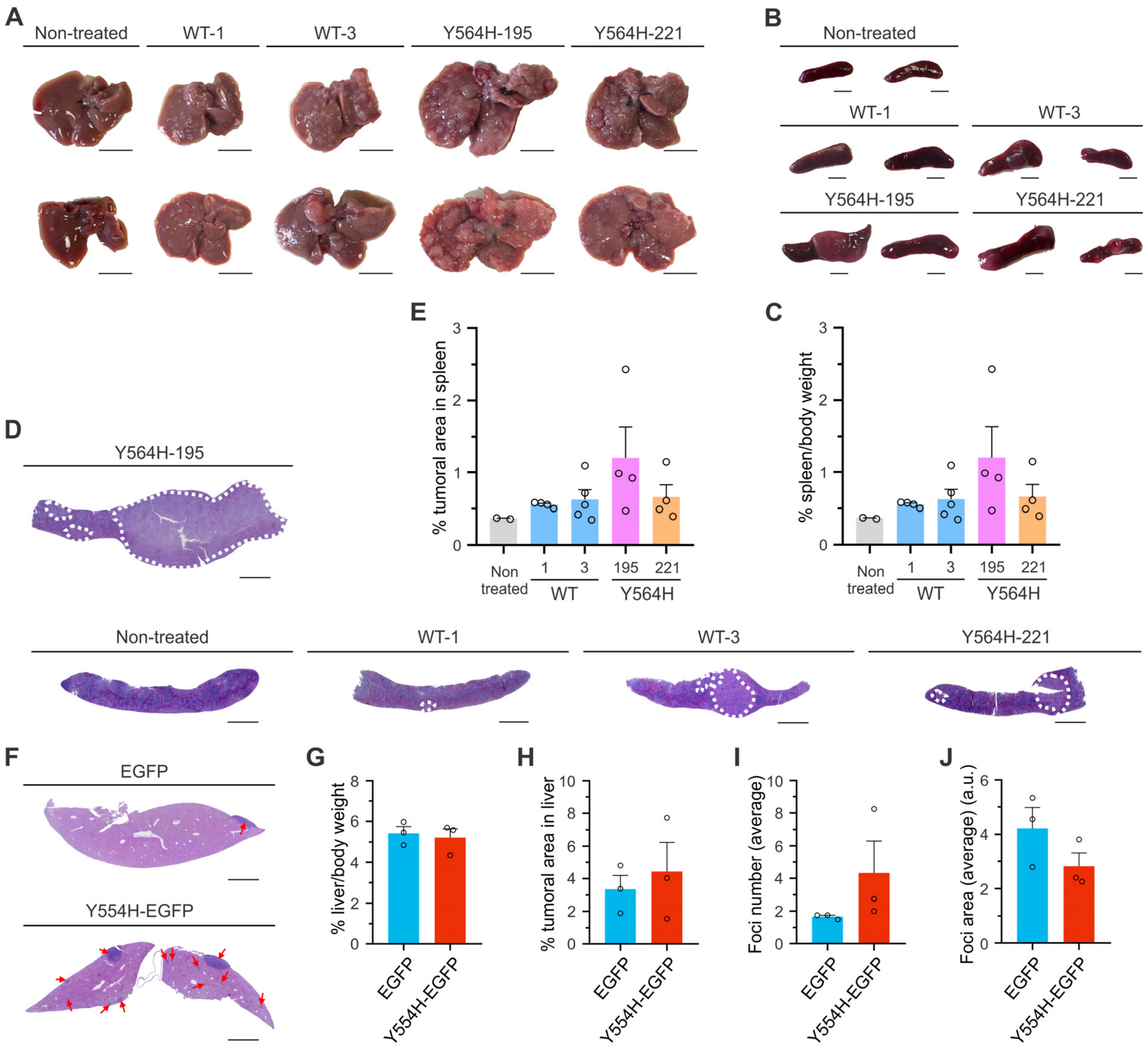

Figure S5. Analysis of tumor growth and metastasis in the liver and spleen of BALB/c mice injected with A20-C3G-Y564H cells, A20-C3G-Y554H-EGFP cells or their corresponding control cells. A total of ~$5x10^5$ A20 cells were injected into the tail vein of BALB/c mice, and tumors were allowed to grow for 21 days. Mice were sacrificed, and liver and spleen were dissected for analysis. (**A**) Images of the livers from mice injected with A20-C3G-WT cells (clones -1 and -3) or A20-C3G-Y564H cells (clones -195 and -221) are shown. The images on the left show the livers of non-injected BALB/c mice, used as controls. Two representative images are provided for each clone, one from each independent experiment. Scale bar: 1 cm. (**B**) Images of the spleens from the same mice whose livers are shown in (**A**). Scale bar: 0.5 cm. (**C**) Bar chart displaying the mean ± SEM of spleen weight as a percentage of total body weight. (**D**) Representative sections of spleens from mice injected with A20-C3G-WT (clones -1 and -3) or A20-C3G-Y564H cells (clones -195 and -221). A spleen section from a

non-treated BALB/c mouse is included as a control. Sections were stained with hematoxylin and eosin, with tumor areas outlined by dotted white lines. Images were captured using a Leica DM6 B microscope with a 5X objective. Scale bar: 0.2 cm. (**E**) Bar chart showing the mean ± SEM of tumor cell area as a percentage of the total spleen section area. Four mice injected with each cell clone were analyzed in four independent experiments. (**F**) Representative liver section from mice injected with A20-EGFP or A20-C3G-Y554H-EGFP cells, 21 days post-injection. Histopathological analysis was performed using hematoxylin and eosin staining. Tumor foci are indicated with red arrows. Scale bar: 0.2 cm. (**G, H**) Percentage of (**G**) liver weight to total body weight (mean ± SEM) and (**H**) tumor cell area as a percentage of the total liver section area (mean ± SEM). (**I, J**) Graphs displaying the mean ± SEM of the (**I**) average foci number and (**J**) average foci area.
