# Supplementary material for: C3G deregulation uncovers a dual role in B-cell lymphoma: tumor suppression and enhanced metastasis via Rap1 and Rac2 signaling
Source: Cell Commun Signal. 2025 Nov 27;24:11. doi: 10.1186/s12964-025-02551-y (PMC12781495; doi:10.1186/s12964-025-02551-y)
Supplement: Supplementary file 2 — Additional file 2. [file 12964_2025_2551_MOESM2_ESM.pdf]

Additional file 2

C3G deregulation uncovers a dual role in B-cell lymphoma: tumor suppression and enhanced metastasis via Rap1 and Rac2 signaling

Running title: C3G dual function in B-cell lymphoma

Alba Morán-Vaquero[1,2,3], Óscar Herranz[1,2,3], Ana Dávila-Hidalgo[1,2,3], Antonio Rodríguez-Blázquez[1,2,3], Cristina Fernández-Infante[1,2,3], Ignacio García-Tuñón[1,2,4], Elena Vuelta[1,2,4], Femke van der Meer[5], Coert Margadant[5], Carmen Guerrero[1,2,3]* and José M. de Pereda[1]*

[1]Centro de Investigación del Cáncer, Consejo Superior de Investigaciones Científicas (CSIC), Universidad de Salamanca, 37007 Salamanca, Spain.
[2]Instituto de Investigación Biomédica de Salamanca (IBSAL), Salamanca, Spain.
[3]Departamento de Medicina, Universidad de Salamanca, Salamanca, Spain.
[4]Departamento de Biomedicina y Biotecnología. Universidad de Alcalá, Alcalá de Henares, Spain.
[5]Institute of Biology, Leiden University, Gorleaus Building, Einsteinweg 55, 2333 CC Leiden, The Netherlands.

*Correspondence and equal contribution: C. Guerrero and JM. de Pereda, Centro de Investigación del Cáncer, Campus Unamuno s/n, Salamanca, Spain. Tel.: +34 923294801; e-mail: cguerrero@usal.es; jm.depereda@csic.es

Supplementary figures

Figure S5

Uncropped Western blots of Figure 2E

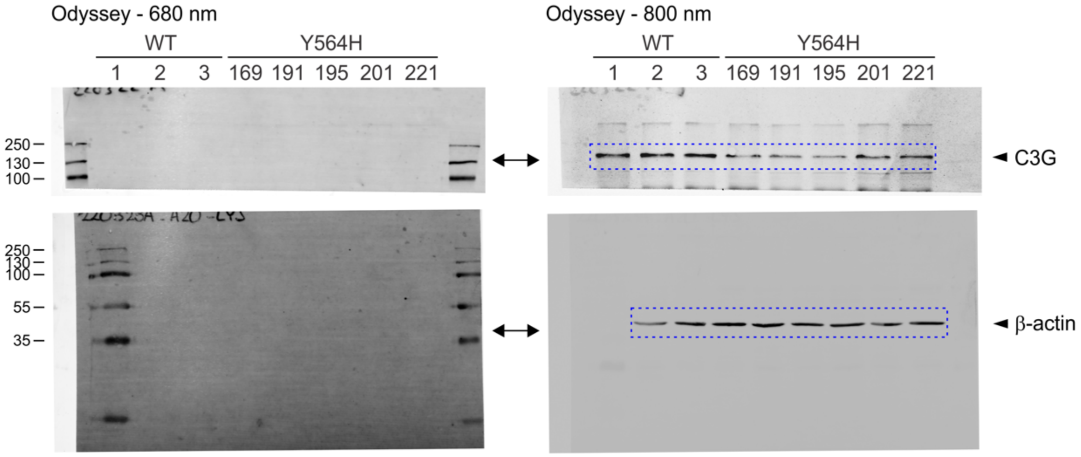

Uncropped Western blots of Figure 2F

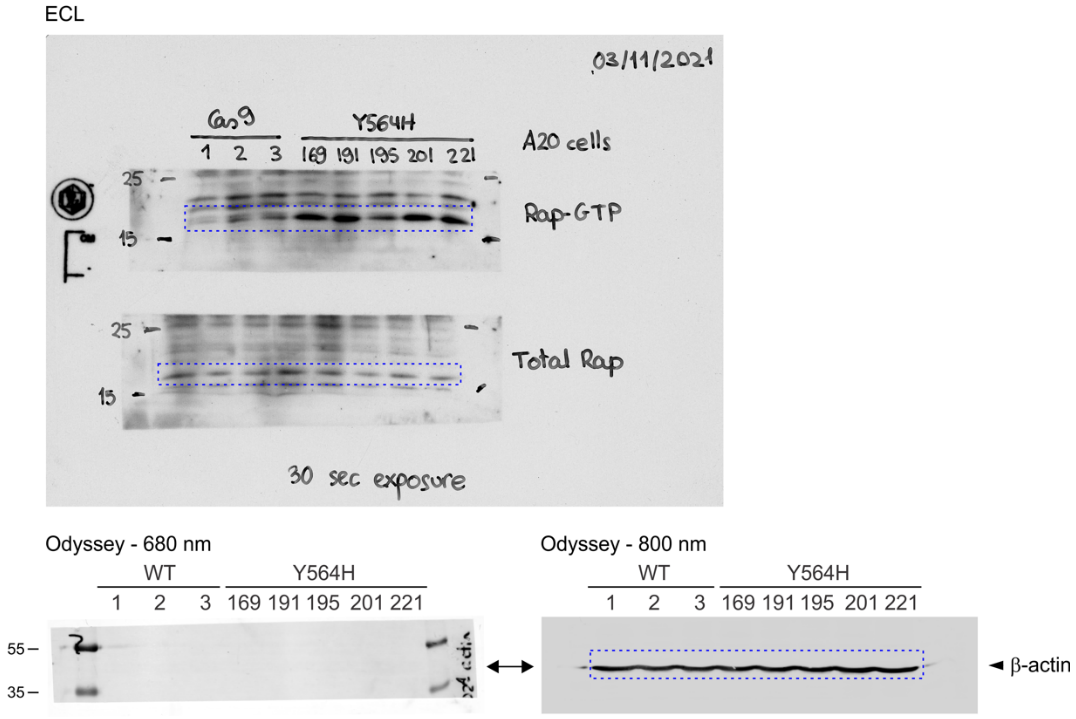

Figure S5 (continuation)

Uncropped Western blots of Figure 2G

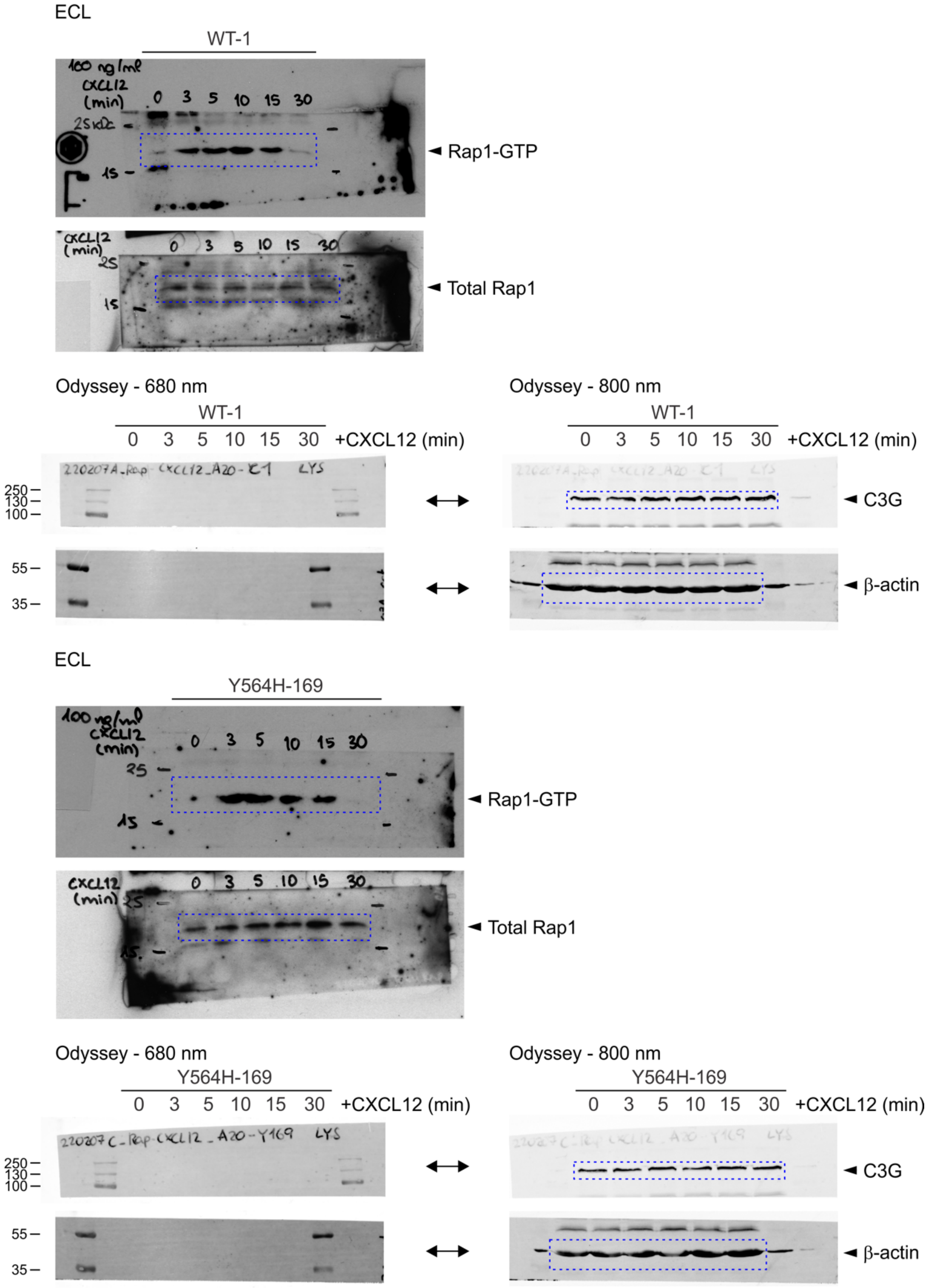

Figure S5 (continuation)

Uncropped Western blots of Figure 2H

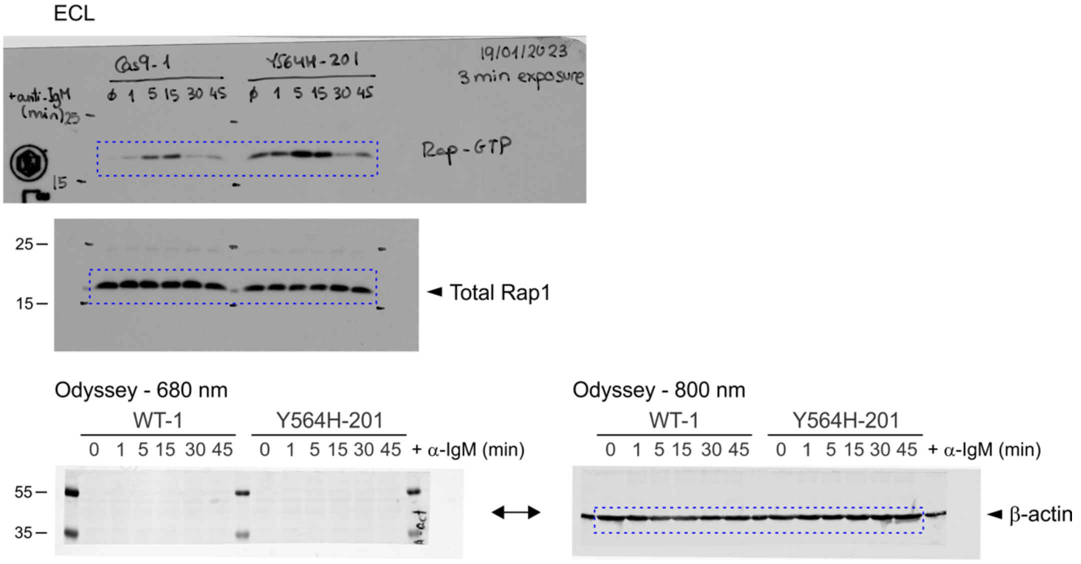

Figure S5. Uncropped images of the western blots shown in Figure 2 of the main manuscript. The corresponding figure and panels from the main manuscript are indicated, along with the molecular weight markers and the channel used for image acquisition with the Odyssey system. Blots developed using electrochemiluminescence (ECL) are indicated.

Figure S6

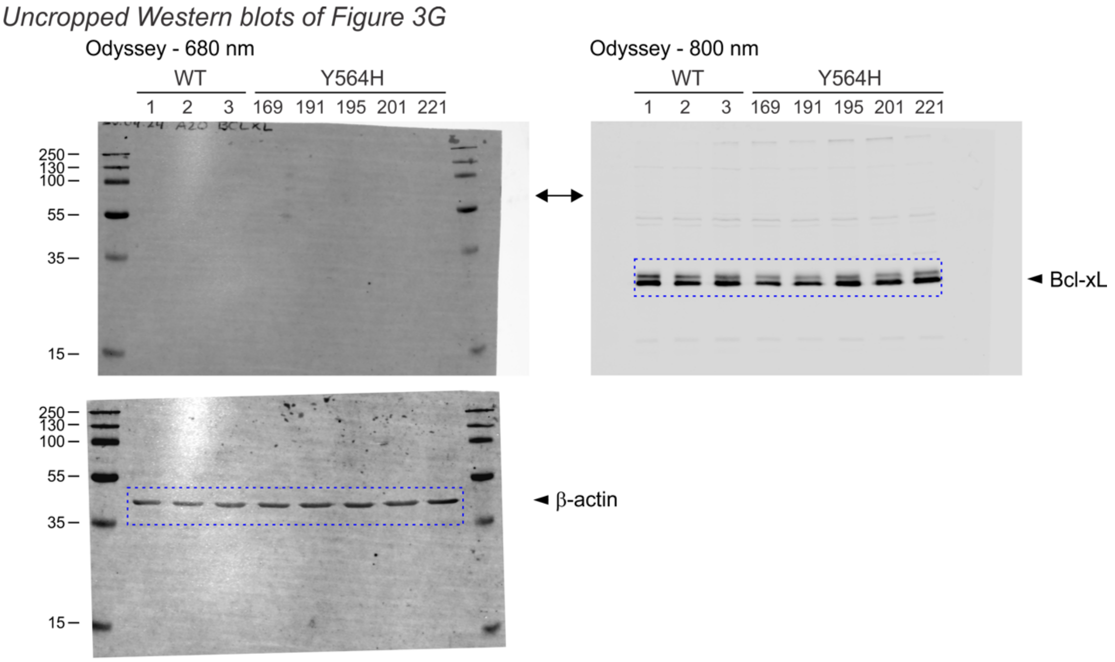

Figure S6. **Uncropped images of the western blot shown in Figure 3 of the main manuscript**. The corresponding figure and panel from the main manuscript are indicated, along with the molecular weight markers and the channel used for image acquisition with the Odyssey system.

Figure S7

Uncropped Western blots of Figure 4A

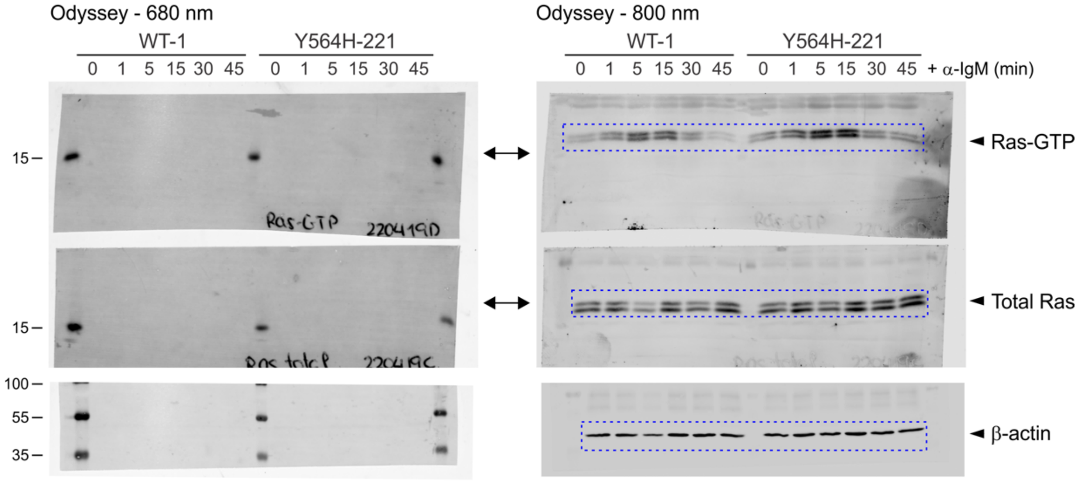

Uncropped Western blots of Figure 4C

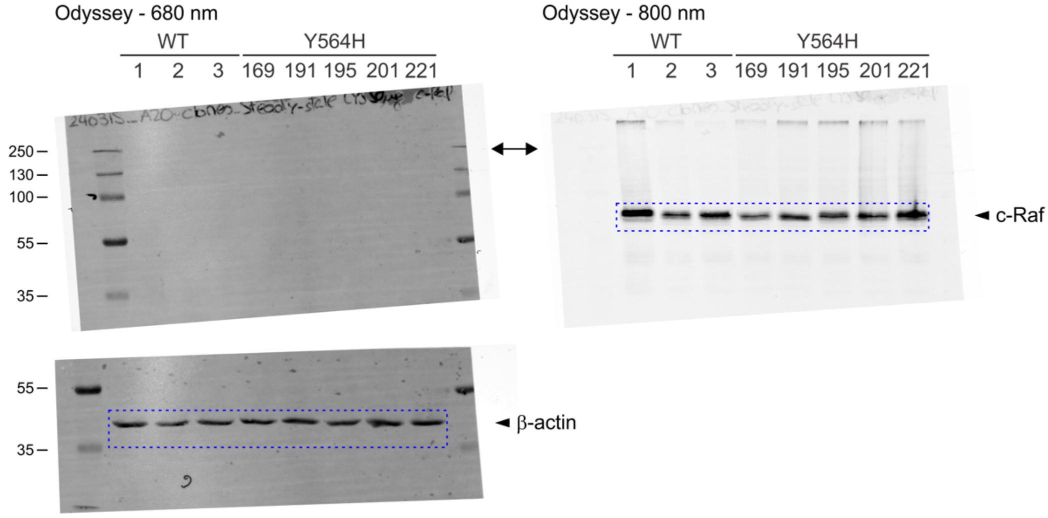

Figure S7 (continuation)

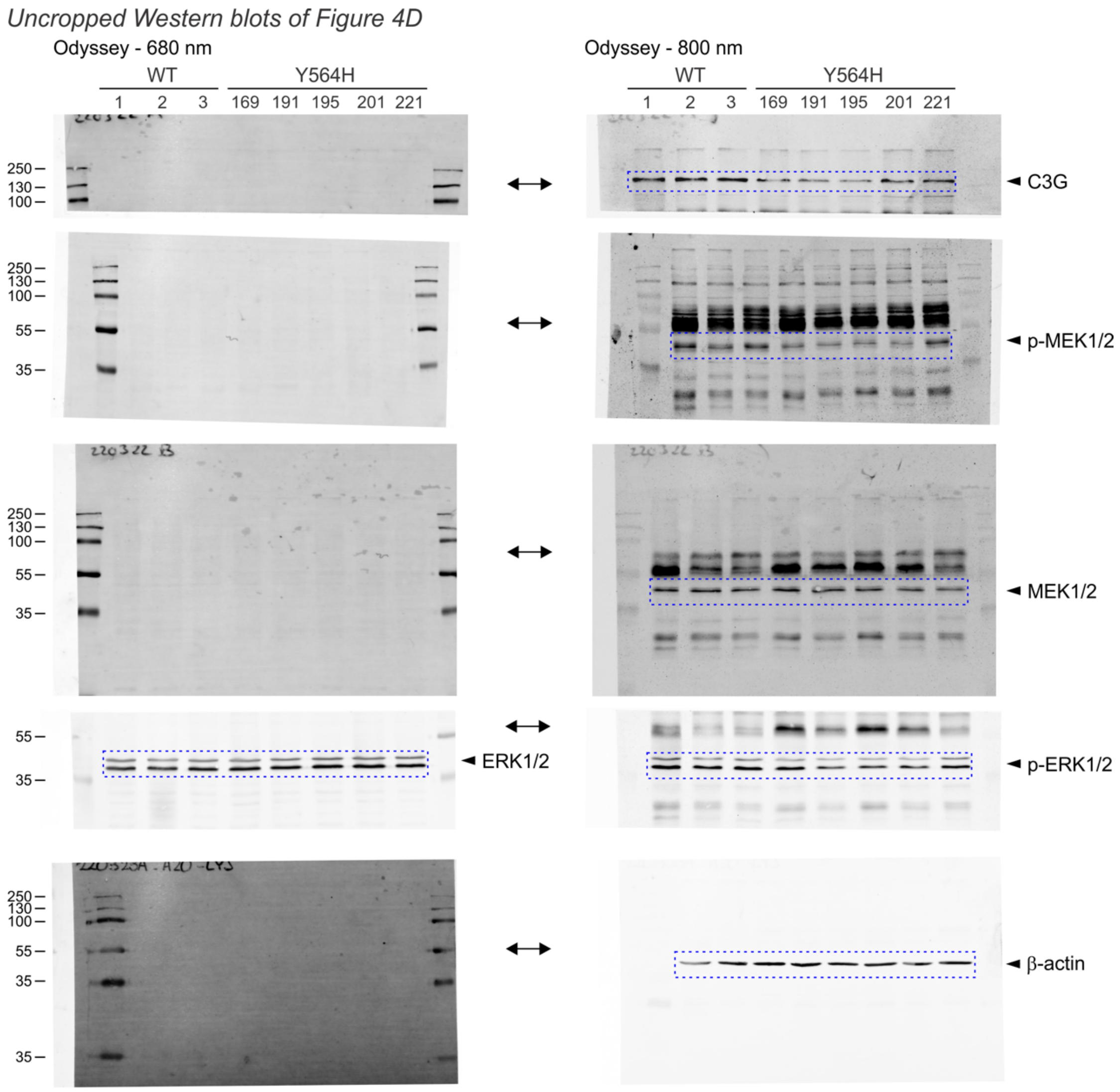

Figure S7 (continuation)

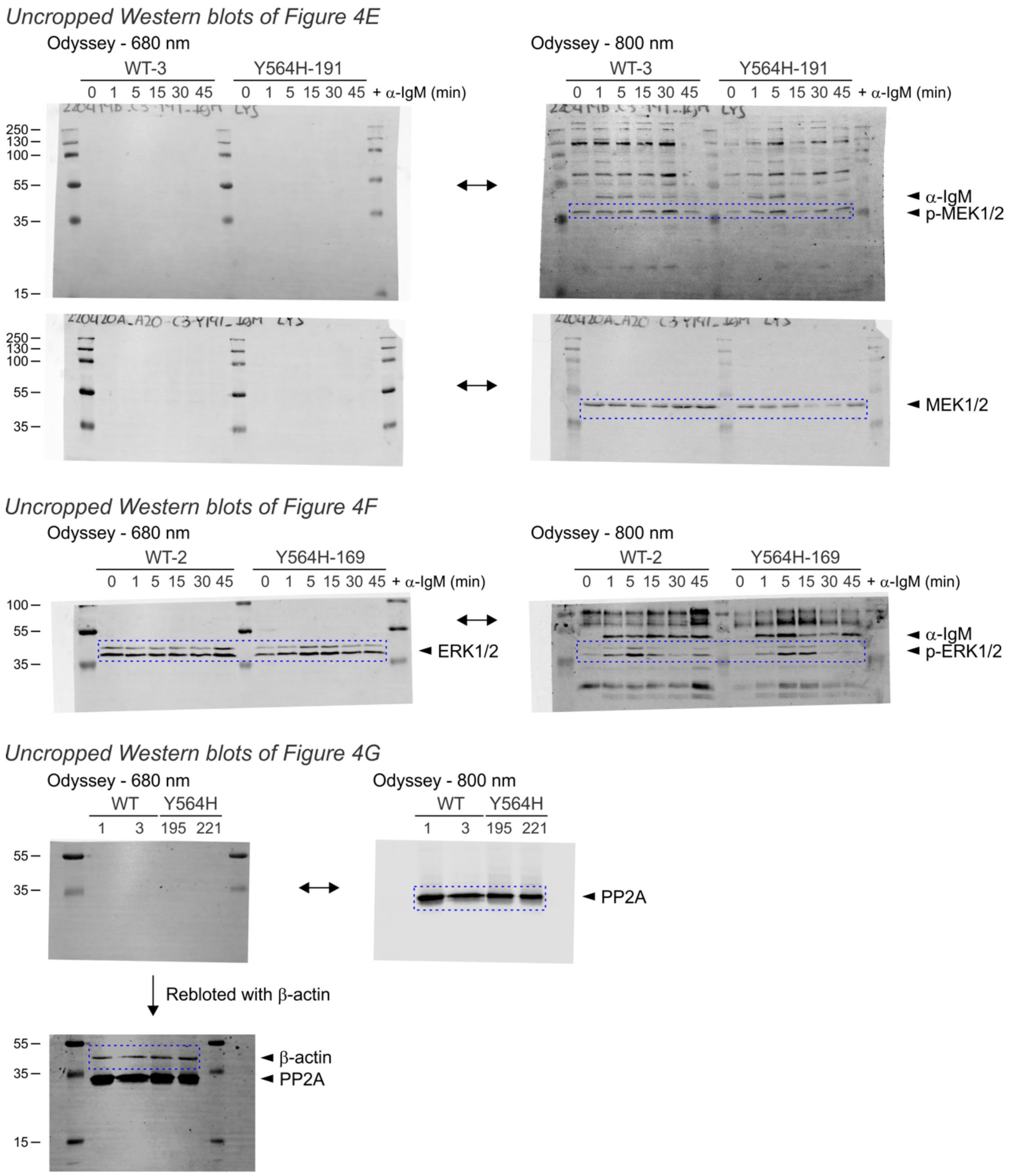

Figure S7. **Uncropped images of the western blots shown in Figure 4 of the main manuscript**. The corresponding figure and panels from the main manuscript are indicated, along with the molecular weight markers and the channel used for image acquisition with the Odyssey system. In the case of the anti-phospho-MEK1/2 and anti-MEK1/2 antibodies, forced exposure was required due to the low quality of these antibodies.

Figure S8

Uncropped Western blots of Figure 5D

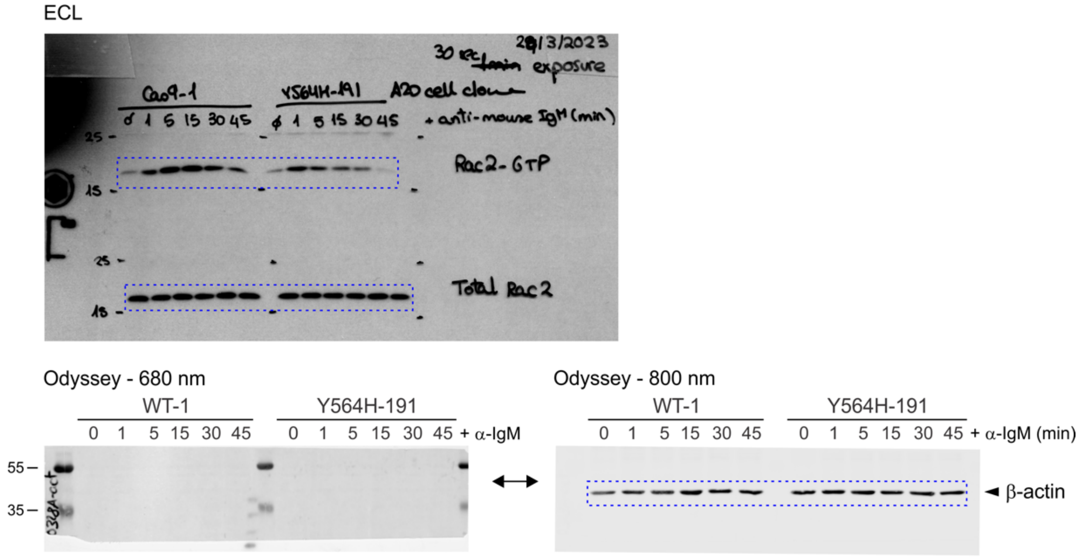

Uncropped Western blots of Figure 5E

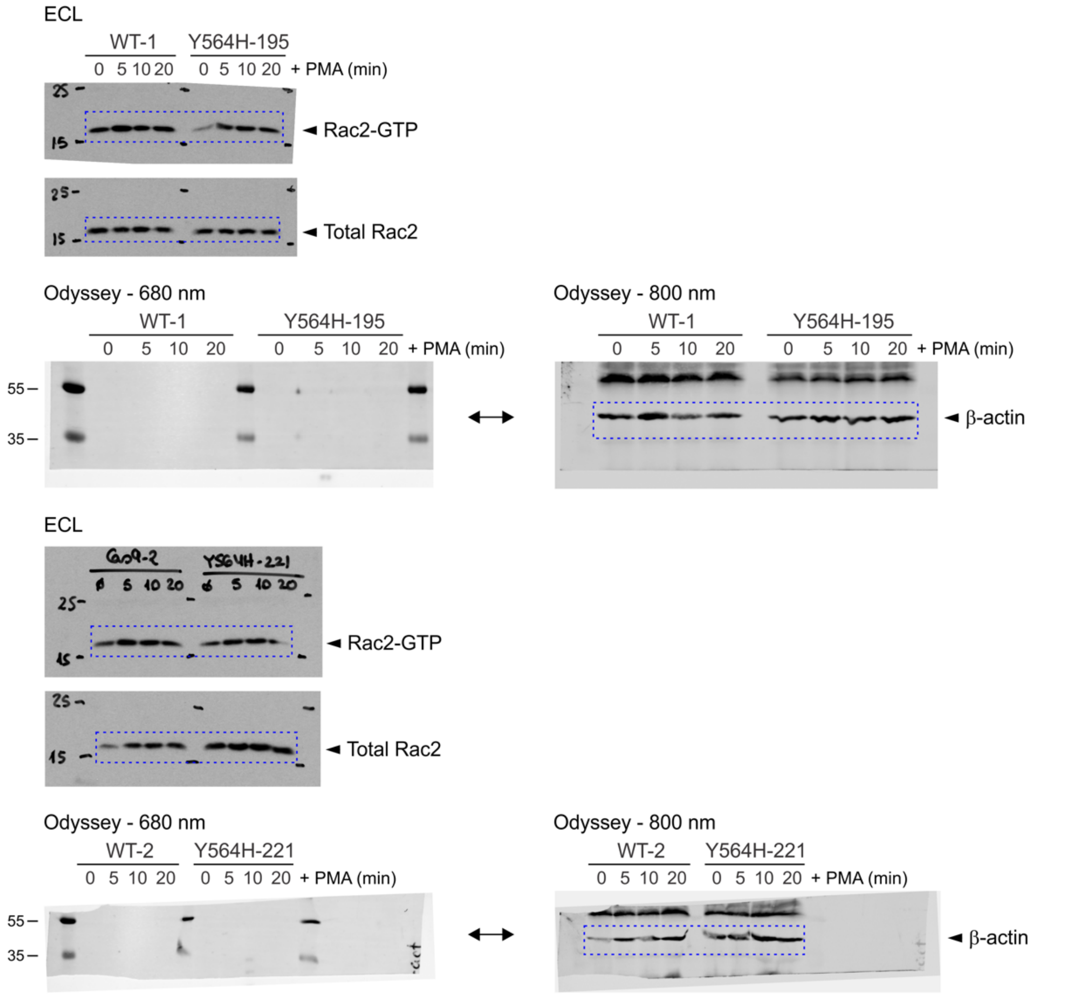

Figure S8 (continuation)

Uncropped Western blots of Figure 5F

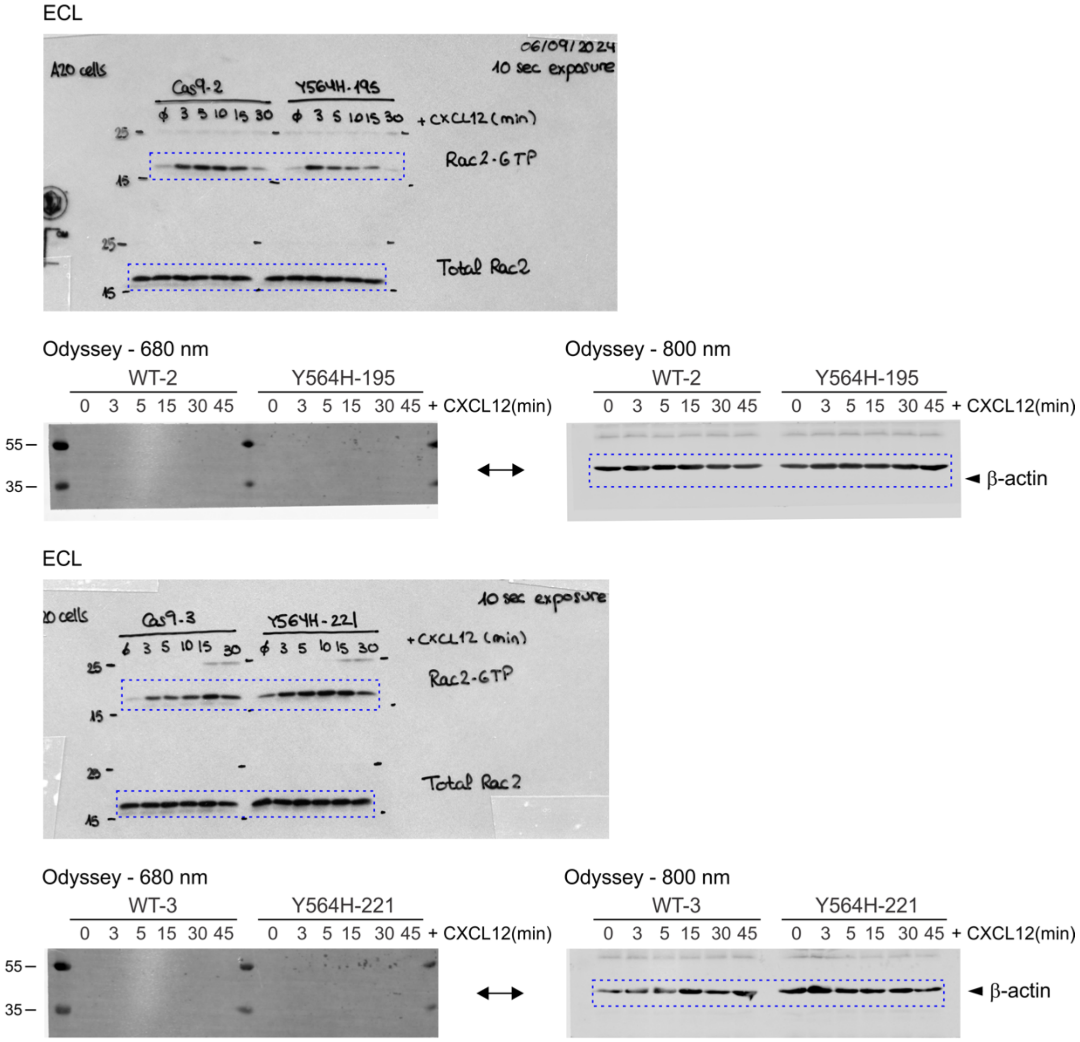

Uncropped Western blots of Figure 5G

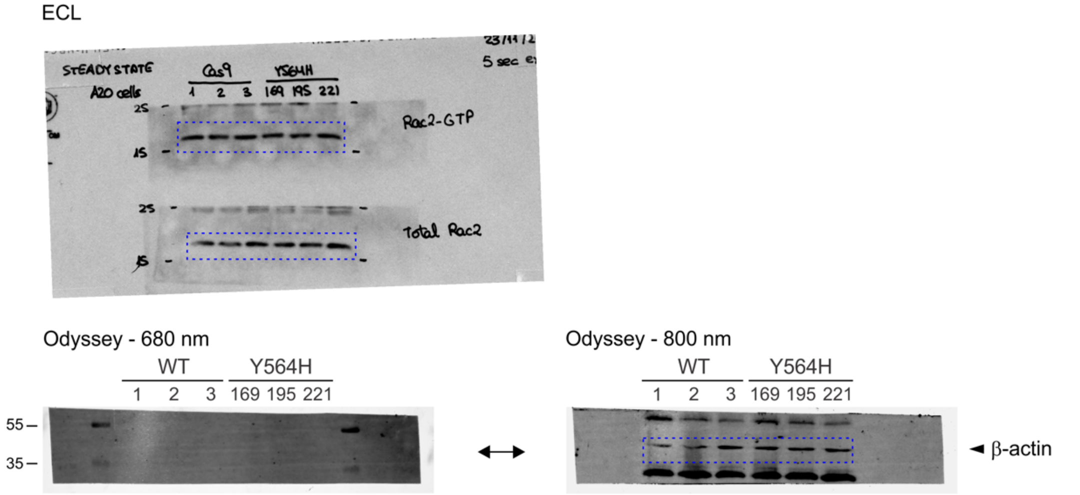

Figure S8. **Uncropped images of the western blots shown in Figure 5 of the main manuscript**. The corresponding figure and panels from the main manuscript are indicated,

along with the molecular weight markers and the channel used for image acquisition with the Odyssey system. Blots developed using electrochemiluminescence (ECL) are indicated. Most of the antibodies used were monoclonal; consequently, some panels display a ~50 kDa band corresponding to endogenous immunoglobulins, as A20 are mouse B cells.

Figure S9

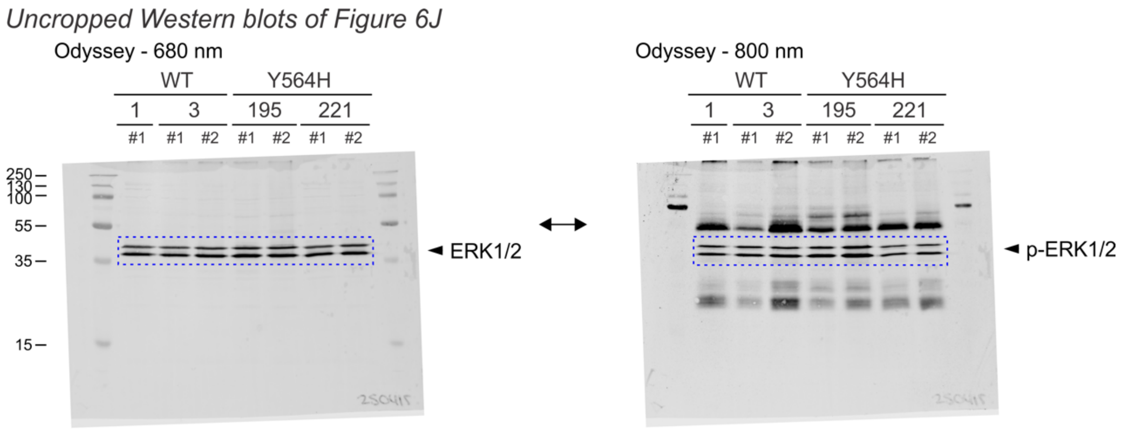

Figure S9. **Uncropped images of the western blots shown in Figure 6 of the main manuscript**. The corresponding figure and panel from the main manuscript are indicated, along with the molecular weight markers and the channel used for image acquisition with the Odyssey system. The anti-phospho-ERK1/2 antibodies used were monoclonal; consequently, the corresponding panel shows a ~50 kDa band derived from endogenous immunoglobulins, as A20 cells are of murine B cell origin.

Figure S10

Uncropped Western blots of Figure S3B

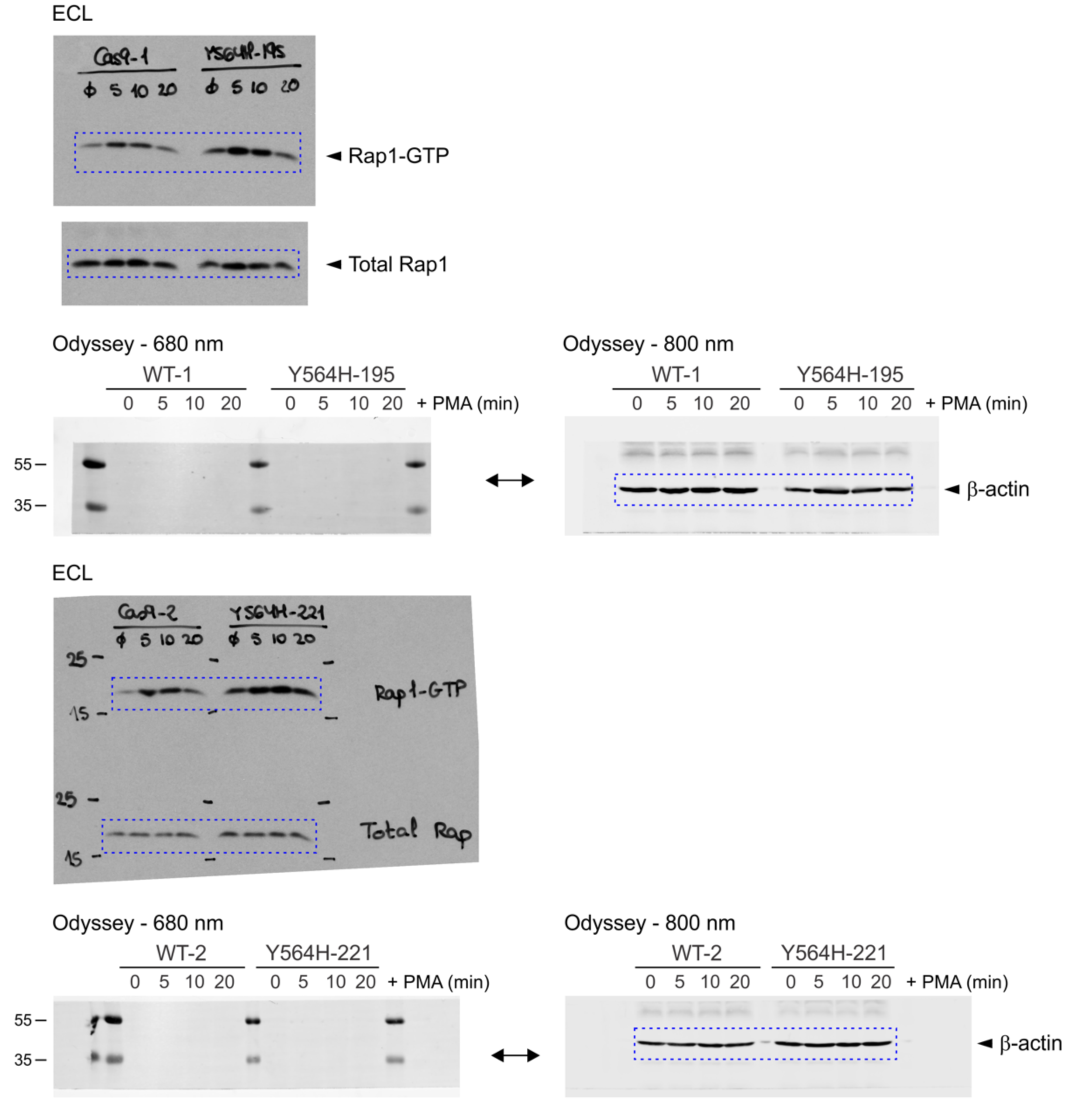

Uncropped Western blots of Figure S3D

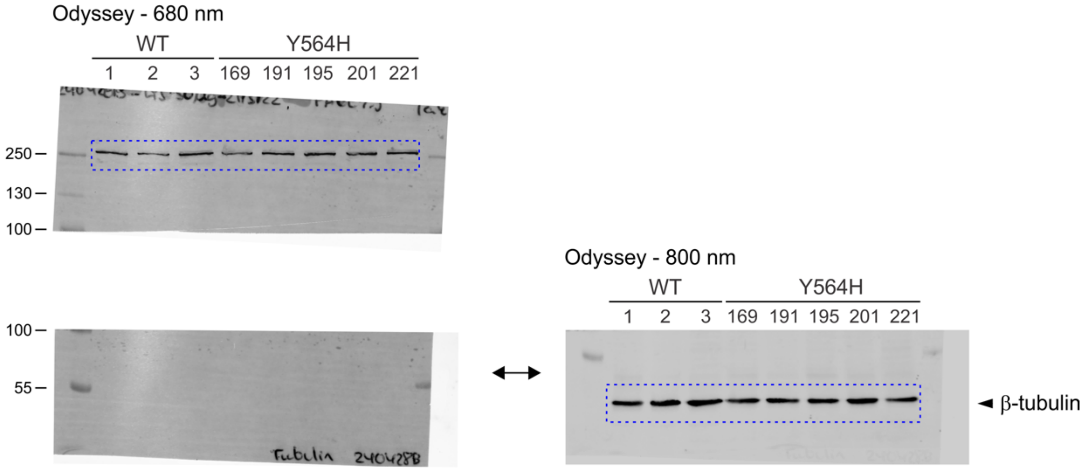

Figure S10 (continuation)

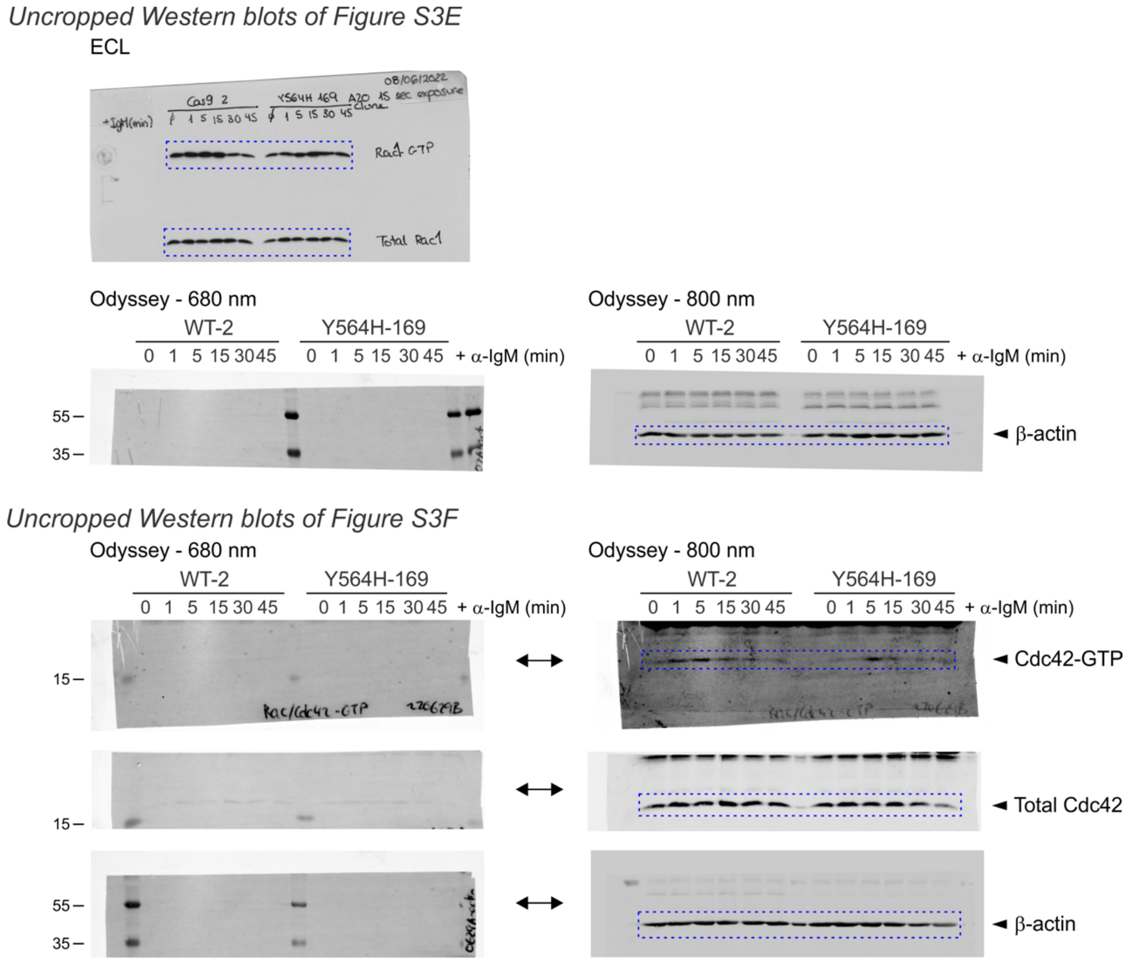

Figure S10. Uncropped images of the western blots shown in Figure S3 of Additional file 1. The corresponding figure and panel from Figure S3 in Additional file 1 are indicated, along with the molecular weight markers and the channel used for image acquisition with the Odyssey system. Blots developed using electrochemiluminescence (ECL) are indicated. Most of the antibodies used were monoclonal; consequently, some panels display a ~50 kDa band corresponding to endogenous immunoglobulins, as A20 are mouse B cells.
